# Neutrophil extracellular traps - a potential trigger for the development of thrombocytopenia during extracorporeal membrane oxygenation

**DOI:** 10.3389/fimmu.2024.1339235

**Published:** 2024-02-21

**Authors:** Moritz Haus, Maik Foltan, Alois Philipp, Thomas Mueller, Michael Gruber, Maximilian P. Lingel, Lars Krenkel, Karla Lehle

**Affiliations:** ^1^ Department of Cardiothoracic Surgery, University Hospital Regensburg, Regensburg, Germany; ^2^ Department of Internal Medicine II, University Hospital Regensburg, Regensburg, Germany; ^3^ Department of Anesthesiology, University Hospital Regensburg, Regensburg, Germany; ^4^ Regensburg Center of Biomedical Engineering, Ostbayerische Technische Hochschule, Regensburg, Germany

**Keywords:** neutrophil extracellular traps (NET), immunoflorescence, thrombocytopenia, extracorporeal membrane oxygenation (ECMO), sepsis, immunothrombosis, coagulation disorder

## Abstract

Neutrophil extracellular traps (NETs) have recently emerged as a potential link between inflammation, immunity, and thrombosis, as well as other coagulation disorders which present a major challenge in the context of extracorporeal membrane oxygenation (ECMO). By examining blood from ECMO patients for NETs and their precursors and correlating them with clinical and laboratory biomarkers of coagulation and inflammation, this study aims to evaluate the association between the presence of NETs in the bloodstream of ECMO patients and the development of potentially severe coagulation disorders during ECMO therapy. Therefore, blood samples were collected from healthy volunteers (n=13) and patients receiving veno-venous (VV) ECMO therapy (n=10). To identify NETs and their precursors, DNA and myeloperoxidase as well as granulocyte marker CD66b were visualized simultaneously by immunofluorescence staining in serial blood smears. Differentiation of DNA-containing objects and identification of NETs and their precursors was performed semiautomatically by a specific algorithm using the shape and size of DNA staining and the intensity of MPO and CD66b signal. Neutrophil extracellular traps and their precursors could be detected in blood smears from patients requiring VV ECMO. Compared to volunteers, ECMO patients presented significantly higher rates of NETs and NET precursors as well as an increased proportion of neutrophil granulocytes in all detected nucleated cells. A high NET rate prior to the initiation of ECMO therapy was associated with both increased IL-6 and TNF-α levels as an expression of a high cytokine burden. These patients with increased NET release also presented an earlier and significantly more pronounced decrease in platelet counts and ATIII activity following initiation of therapy compared with patients with less elevated NETs. These findings provide further indications for the development of immune-mediated acquired thrombocytopenia in ECMO patients.

## 1 Introduction

Extracorporeal membrane oxygenation (ECMO) has emerged as an important therapeutic modality for critically ill patients with acute and severe respiratory or cardiac failure (1–4). Since its first use, continuous improvement of the ECMO devices used as well as the care of ECMO patients has resulted in major advances, especially regarding the recognition and treatment of ECMO related complications. Nevertheless, coagulation-associated technical complications, such as clot formation or critical bleeding, remain a major cause of mortality and morbidity during ECMO therapy (5) leading to device malfunction and the necessity for high-risk system replacement in 31-51% of all patients on veno-venous (VV) ECMO (3, 6, 7).

Several contributing factors have been identified as leading causes for the development of hemorrhagic diathesis on ECMO. These include the synergistic effects of platelet and plasma dilution at therapy initiation, therapeutic anticoagulation, and acquired platelet and plasmatic coagulation disorders (8, 9). In this regard, acquired trombobocyte dysfunction, acquired von Willebrand syndrome and hyperfibrinolysis with consecutive consumption coagulopathy represent the most important pathomechanisms (8–10). Concerning thrombus formation, various thrombogenic factors have been identified in ECMO treatment. In particular, the large contact area of the artificial ECMO circuit with the patient’s blood as well as high shear forces within the device seem to have an influence on the activation of platelets, leukocytes and the plasmatic coagulation cascade (9, 11–14). This interplay of leukocytes, platelets and plasmatic coagulation in thrombus formation on different biomaterials has been described (13, 15–17) and adherent leukocytes could be detected on the gas exchange membranes of ECMO oxygenators as a sign of immune-mediated thrombus formation (18–20).

With the description of neutrophil extracellular traps (NETs) as part of the innate immune response of neutrophil granulocytes by Brinkmann et al. in 2004 (21) and subsequent extensive research, a link between neutrophil granuolocytes as the largest fraction of leukocytes and the formation of thrombi has been demonstrated (22–26). These NETs are defined as “extracellular [often string like] structures composed of chromatin and granule proteins” (21, 27) of neutrophil granulocytes such as myeloperoxidase (MPO) or neutrophil elastase. Since then, the process of NETosis has been further unraveled and specific alterations of the neutrophil nucleus have been demonstrated with significant nuclear decondensation prior to ejection of the DNA-protein complex, allowing identification of NET precursors among the neutrophils (27–30). *In vivo*, acute or chronic, local or generalized inflammatory processes are the main inducers of NETosis, so that NETs can be detected in particular in critically ill patients with sepsis or patients with chronic autoimmune diseases such as SLE or rheumatoid arthritis (31–35).

The thrombogenic potential of NETs could be shown frequently (22, 23), thus establishing the new entity of immunothrombosis (24–26). This immune-mediated thrombus formation has been identified in a variety of diseases with thrombus formation, such as myocardial infarction or stroke (36, 37). NET formation on various artificial surfaces (38, 39) as well as shear stress induced NET formation (40) has been demonstrated. Most recently NETs were also detected in thrombi found in ECMO circuits (41), leading to the question of what role NETs play in the development of coagulation-associated complications during ECMO treatment and whether they may be suitable as a biomarker to predict such events.

After NETs could be detected in a wide variety of biomaterials in the past, blood smears seemed to be a promising approach as quick and easy to perform, inexpensive and non-invasive method due to the minimal amount of blood required and the ability to draw blood from the ECMO circuit. A similar approach to assess a patient’s NET status by blood smears has been described earlier in the literature (31, 32).The aim of this study was to detect neutrophil extracellular traps in blood smears of critically ill patients receiving veno-venous ECMO therapy and to evaluate the association between the presence of NETs in the bloodstream and the development of potentially severe coagulation disorders during ECMO therapy.

## 2 Materials and methods

### 2.1 Patients and settings

This study was a prospective observational study and approved by the Ethics Committee of University Medical Hospital Regensburg (vote no. 16-101-0322). Written informed consent was obtained from 13 young and healthy volunteers without any symptoms of acute respiratory infection and 10 patients who required VV ECMO due to acute lung failure. From December 2017 to May 2019, blood samples were collected in EDTA tubes before, 2h, 6h, 24h, 48h and 120h after ECMO implantation and in selected cases before and after a system exchange. As many ECMO cannulations were carried out in external hospitals, the blood samples gathered before ECMO initiation by the perfusionist were transported to the laboratory together with the 2h samples after arrival of the patient in the intensive care unit. From the 2h sample onwards, all blood smears were prepared within 20-30 min by scratching 5µL of blood in a thin layer onto glass slides. The blood smears were dried and stored at -80°C until immunostaining (32). All samples were pseudonymized before further processing to ensure unbiased evaluation.

### 2.2 Positive controls for the detection of NETs and NET precursors

In preceding experiments, neutrophil granulocytes were isolated from the blood of volunteers, adhered to poly-L-lysine coated slides and stimulated with PMA (“positive control”) or remained non-stimulated (“negative control”) as described (21, 27, 28, 30). Positive controls were prepared to assess various staining strategies for the blood smears and as a reference for the detection of NETs and NET precursors. Examples of the positive controls as well as details regarding isolation and stimulation of neutrophils are provided in the supplement.

### 2.3 Immunostaining and fluorescence microscopy

To identify and distinguish between regular nucleated cells, NETs and NET precursors, we used a triple colour fluorescent staining to simultaneously visualize DNA (DAPI, 4´,6-diamidino-2-phenylindole), CD66b and myeloperoxidase (MPO). Blood smears were fixed (paraformaldehyde, 4%, 10min), washed in deionized water and Tris-buffered saline (TBS) and permeabilized with TBS containing 0.025% TritonX100 and 0.025% Tween20. Blocking was performed with TBS with added 1% bovine serum albumin, 2% normal donkey serum, 0.2% coldwater fish gelatine, 0.05% TritonX100 and 0.05% Tween20 for 2h at room temperature.

After washing, blood smears were incubated overnight with primary antibodies [antihuman MPO rabbit polyclonal antibody (DAKO, Hamburg, Germany; 1:2500); antihuman CD66b mouse monoclonal antibody (Biorbyt, Cambridge, UK; 1:600)] at 4°C. After washing, each primary antibody was visualized with a secondary antibody [Alexa-Fluor-594-conjugated donkey antirabbit monoclonal antibody; Alexa-Fluor-488-conjugated donkey antimouse monoclonal antibody (both Dianova, Hamburg; 1:600)] incubation for 60min at room temperature in the dark. After washing, slides were mounted with 4´,6-diamidino-2-phenylindole (DAPI) in Fluoromount-G (SouthernBiotech, Birmingham, AL, USA) (overnight, 4°C, dark). Staining of the PMA-stimulated positive controls was performed following the same protocol, but additional primary antibodies (e.g. against citrullinated histone H3) were tested for their eligibility in a serial analysis of the blood smears (**Supplementary Figures S1–S3**). Isotype control antibodies as well as staining of only the secondary antibodies were used to rule out non-specific bindings.

The stained blood smears were examined with an inverted fluorescence phase-contrast microscope (Keyence BZ-8100E, Neu-Isenburg, Germany) at 200x magnification. After adjusting every field of view manually to an optimum, each of the 3 fluorescence channels was captured as a RGB image with a resolution of 2720x2048 pixels using an integrated CCD sensor. In order to evaluate a representative area of each slide and taking into account the fluctuating number of cells/objects per field of view, 30 randomly predefined, non-overlapping positions on the slide were examined in sequence. All slides were blinded before imaging so that no conclusions could be drawn about the origin of the sample during microscopy.

### 2.4 Identification of objects in blood smears

An automated macro in the open-source image processing software FIJI version 1.52n (U. S. National Institutes of Health, Bethesda, Maryland, USA, https://imagej.nih.gov/ij/) was used to identify objects in the stained blood smears. All RGB images were split in their red, green and blue colour components and converted to an 8-bit grayscale. In order to identify the nucleated objects with DAPI staining, the object contours were first enhanced from the background by adjusting the contrast and applying a filter. By applying a black and white threshold creating a binary image of the object outlines, the DAPI-stained objects could thus be characterized and measured as individual regions of interest (ROIs), which is demonstrated in **Supplementary Figure S4**. Each ROI corresponding to a single object, i.e. a nucleus, NET filament, etc., was then measured in terms of size and shape [area, maximum distance between any two points along the object’s circumference (“maximum feret´s diameter”), aspect ratio of an ellipse fitted around the object]. Before measurement, the frequently blurred edge areas were automatically excluded and obvious artifacts (e.g., overlapping cell nuclei that would have resulted in a distorted size measurement) were corrected manually. To assess each DNA-containing object for the presence of CD66b and MPO, the ROI masks generated by DAPI staining were transferred to the individual grayscale images of the two antibody stainings. Detection of CD66b and MPO was achieved by measuring the maximum intensity of the respective staining within an ROI.

### 2.5 Identification and quantification of nuclei, NETs and their precursors

To distinguish between regular nuclei, NETs, and NET precursors, an automated algorithm (**Supplementary Figure S5**) was developed. The cut-off values for each measured parameter used to classify the individual objects were determined based on a receiver operating characteristic (ROC) analysis using randomly selected and manually evaluated images (n=100) as a reference for the object categories (regular nuclei, NETs, NET precursors, artifacts).

All DNA-containing objects were assigned to their respective categories based on their shape and size and colocalization of CD66b and MPO. Objects with an area of 39µm² to 134µm² were identified as granulocyte nuclei by a positive CD66b signal (42–44) and were further differentiated into neutrophils (MPO positive) and eosinophils (MPO negative) based on MPO staining (45–47). Basophils were neglected. An exclusively positive MPO signal and a nuclear area comparable to that of granulocytes classified monocytes (45, 48). Lymphocytes were defined by their round nuclei with an area of 39µm² to 139µm² and the absence of CD66b and MPO. NETs are “extracellular [often string like] structures composed of chromatin and granule proteins” (21, 27). Therefore, elongated (determined by an aspect ratio of ≥2.15 or a feret’s diameter of ≥19µm), DNA-containing objects with a positive signal for CD66b and/or MPO as a sign of neutrophil origin were categorized as NETs (46, 47). The swollen and decondensed nuclei of NET precursors (27–30) were identified by their enormous size of ≥135µm² with a rather roundish to oval nuclear shape and positive staining for CD66b and/or MPO. Objects that could not be assigned to any of the object categories (e.g., very small objects or very large objects without CD66b and MPO detection) were classified as artifacts. The specific thresholds for size, shape and fluorescent staining of each object category are presented in **Supplementary Figure S5**.

We analyzed the proportion of neutrophils, eosinophils, monocytes and lymphocytes in all regular nucleated cells. The proportion of NETs (NET rate) was specified as the number of NETs relative to the sum of neutrophils, NETs and precursors. The proportion of precursors (precursor rate) was specified as the amount of decondensed nuclei relative to the sum of neutrophils, NETs and precursors.

### 2.6 Defining subgroups based on the NET rate at the time of ECMO initiation

On the basis of the NET rate measured in the blood smears of the controls, a cutoff value was defined according to the 3 sigma rule (mean + 3 x standard deviation) (49), which was used to classify each patient as “HIGH-NET” or “LOW-NET” at the time of ECMO therapy initiation.

### 2.7 Data collection and statistical analysis

Patient data, including the routinely performed laboratory diagnostics for all patients, were collected prospectively (Regensburg ECMO database) and pseudonymized to the investigators. All laboratory diagnostics were performed in the Institute for Clinical Chemistry and Laboratory Medicine of the University Hospital Regensburg using EDTA blood (complete blood count), lithium heparin plasma (CRP, LDH, creatinine, free hemoglobin), citrate plasma (INR, aPTT, D-dimers, fibrinogen, ATIII activity) and serum (IL-6, TNF-α). IBM SPSS Statistics v.29.0 (IBM Corporation, Armonk, USA) was used for statistical evaluation. Data are presented as median and interquartile ranges (IQR). The displayed boxplot graphs visualize median and interquartile range (IQR), the whiskers represent minimum and maximum values. In addition, the individual data points are displayed as dots. Testing for differences in central tendency was performed by applying the Mann-Whitney U test to independent samples and the Wilcoxon signed-rank test to dependent samples. Multiple dependent samples were examined by applying the Friedman test followed by a post-hoc analysis with Dunn-Bonferroni correction for multiple testing. Analysing for correlation was performed using Spearman’s rank correlation. Except for the post-hoc analysis with Dunn-Bonferroni correction, where only the asymptotic significance could be calculated, all p-values displayed correspond to the exact 2-sided significance. A p-value of less than or equal to 0.05 was considered statistically significant.

## 3 Results

### 3.1 Detection and differentiation of NETs, their precursors and other nucleated cells

Isolated and PMA-stimulated neutrophils served as a reference for the detection of NETs and their precursors. All isolated neutrophils presented a positive CD66b signal. Approximately 90-120 min after stimulation with PMA, the onset of nuclear decondensation with progressive nuclear enlargement and loss of its lobulation was observed. Prior to decondensation, citrullinated H3 (citH3) could be detected in the stimulated nuclei to a large extent (**Supplementary Figure S1**). Following decondensation, NET formation with expulsion of neutrophil DNA and granulocytic proteins was observed at first after 120-180 min. In the resulting string-like NET filaments, CD66b and MPO could be reliably detected in parallel to DNA (**Supplementary Figures S2, S3**), however, staining for citH3 revealed enormous fluctuations in terms of intensity and staining pattern within the individual NET filaments (**Supplementary Figure S2**).

**Figure 1:**
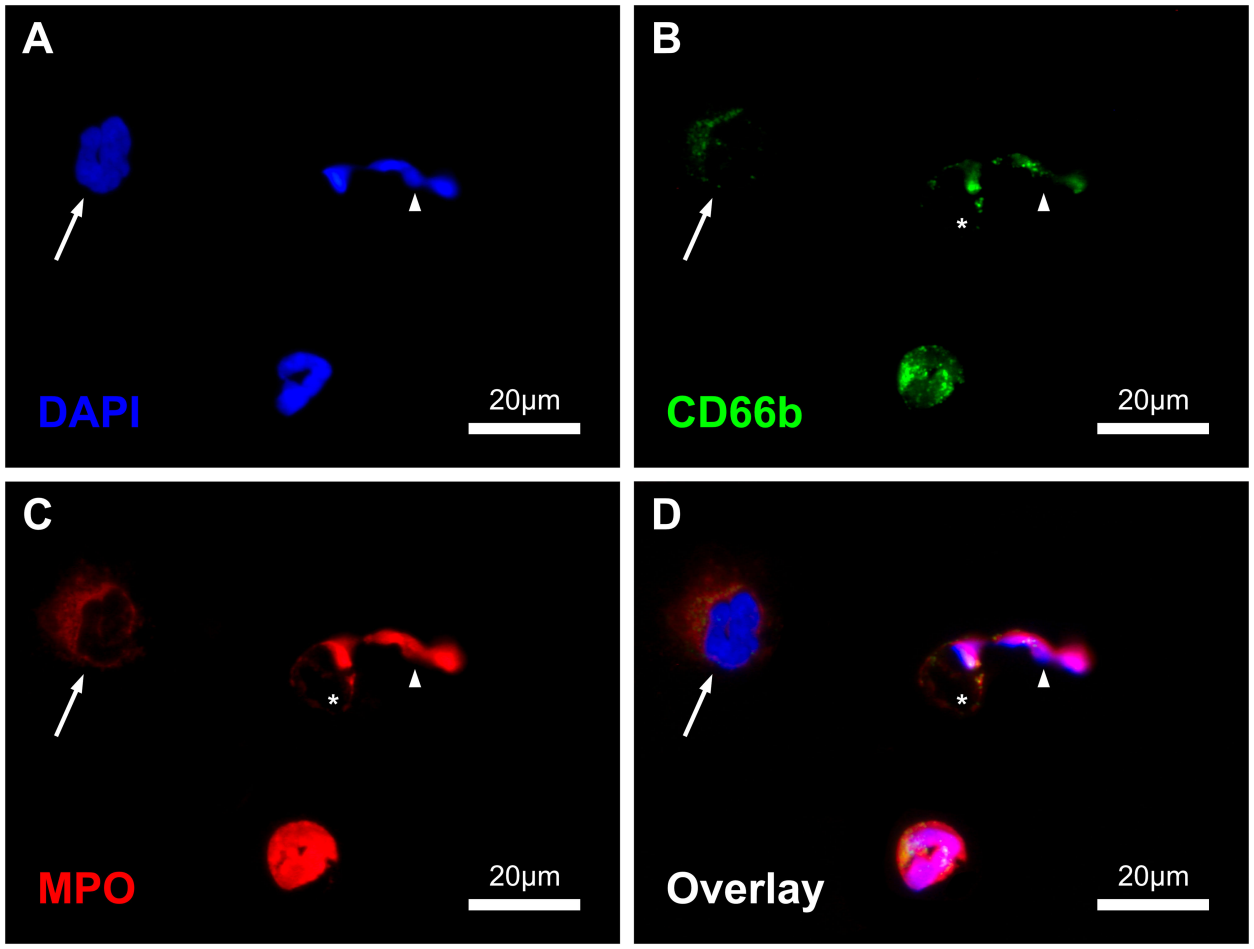
Immunofluorescence-stained image of the blood smears with displayed channels for intercalating DAPI-DNA-staining **(A)**, CD66b- **(B)** and MPO-Immunostaining **(C)** as well as the combined overlay **(D)**. In the image section one NET (arrowhead) with residual cell body (asterisk, *), one NET precursor with enlarged and decondensed nucleus (arrow) and one regular neutrophil granulocyte are shown. Scale: 20µm.

As these findings from the positive controls were also observed in blood smears, a triple staining strategy based on DNA, CD66b and MPO was established for the subsequent serial analysis. With the applied methodology, the different healthy cell types (neutrophil and eosinophil granulocytes, monocytes and lymphocytes), as well as NETs and their precursors could be reliably detected and differentiated within the blood smears (**Figure 1**; **Supplementary Figure S6**).

### 3.2 Study population, laboratory and ECMO run data

Data were collected from 10 patients who required VV ECMO mainly due to bacterial (30%) and viral (50%) pneumonia. The obtained data were already presented with respect to the groups formed on the basis of the NET rate before the start of ECMO. Baseline characteristics are summarized in **Table 1**. The median age of 8 male and 2 female patients was 57 (48/61) years with elevated inflammatory baseline data. The median SOFA score was 11 (10/14), one patient required renal replacement therapy prior to ECMO-therapy. Due to a change in the standard anticoagulation regime for patients receiving VV ECMO in our ECMO center in 2017, 50% of our patients were anticoagulated with heparin, the other 50% with argatroban (51). The decision about the type of anticoagulation was based on clinical considerations and was not considered for study inclusion.

**Table 1:** Baseline characteristics and laboratory data from all patients (n=10) and from the two NET-rate-derived subgroups [HIGH-NET (n=4) and LOW-NET (n=6)] prior to the beginning of ECMO therapy.

	All	HIGH-NET	LOW-NET	p-value
**Patients (n)**	10	4	6	
**Male gender (n, %)**	8, 80	3, 75	5, 83	1.000
**Age (years)**	57 (48/61)	55 (51/60)	60 (46/61)	0.871
**BMI**	27.8 (24.7/30.3)	27.7 (24.1/30.3)	27.8 (24.7/30.3)	0.914
**Ventilation pre ECMO (days)**	1 (0/11)	6 (1/12)	1 (0/2)	0.338
Cause of respiratory failure				
Bacterial pneumonia (n, %) Viral pneumonia (n, %) Post-operative ALF (n, %) Aspiration pneumonia (n, %)	3, 30 5, 50 1, 10 1, 10	2, 50 1,25 0, 0 1, 25	1, 17 4, 67 1, 17 0, 0	
**CPR prior to ECMO (n, %)**	2, 20	0, 0	2, 33	0.467
**CVVHF prior to ECMO (n, %)**	1, 10	0, 0	1, 17	1.000
**CVVHF during ECMO (n, %)**	3, 30	1, 25	2, 33	1.000
**SOFA-Score** ≤ 10 (n, %) > 10 (n, %)	11 (10/14) 4, 40 6, 60	11 (9/13) 2, 50 2, 50	12 (10/14) 2, 33 4, 67	0.524
**Positive DIC-Score** (n, %)	3, 30	2, 50	1, 17	0.500
**WBC (/nL)**	13.8 (10.2/14.3)	13.3 (10.2/14.3)	13.8 (10.2/14.9)	0.719
**CRP (mg/L)**	256 (178/293)	155 (81/232)	293 (256/318)	0.111
**Interleukin-6 (pg/mL)**	598 (275/4709)	8069 (2659/105566)	401 (211/587)	**0.019**
**TNF-α (pg/mL)**	36 (19/48)	64 (42/262)	21 (12/32)	**0.038**
**INR**	1.1 (1.0/1.2)	1.1 (1.0/1.2)	1.2 (1.0/1.3)	0.448
**aPTT (seconds)**	39 (35/42)	42 (35/50)	35 (35/42)	0.619
**Platelets (/nL)**	223 (168/256)	173 (79/246)	228 (174/269)	0.352
**D-dimers (mg/L)**	7 (4/11)	10 (6/12)	6 (4/8)	0.457
**Fibrinogen (g/L)**	504 (412/573)	444 (354/547)	538 (422/573)	0.476
**ATIII activity (%)**	56 (49/72)	50 (38/72)	60 (50/72)	0.610
**Free hemoglobin (mg/L)**	30 (27/45)	53 (19/135)	29 (27/42)	0.476
**LDH (U/L)**	331 (259/493)	278 (202/395)	385 (544/320)	0.352
**Creatinine (mg/dL)**	1.0 (0.6/1.7)	0.9 (0.6/2.1)	1.1 (0.6/1.7)	0.648

Data are displayed as absolute quantities n with proportions or as median (IQR, interquartile range); BMI, Body mass index; ALF, acute lung failure; CPR, Cardiopulmonary resuscitation; CVVHF, continuous venovenous hemofiltration; SOFA, Sequential Organ Failure Assessment; DIC, disseminated intravasal coagulation [JAAM 2016 criteria, (50)]. WBC, white bloodcell count; CRP, C-reactive protein; TNF-α, tumor necrosis factor alpha; INR, International normalized ratio; aPTT, activated partial thromboplastin time; ATIII, Anti-thrombin III; LDH, lactate dehydrogenase.

Different ECMO systems were used, the ECMO run data is presented in **Table 2**. The median time of total ECMO support was 12 (8/17) days. In four patients an exchange of the ECMO system was necessary. Eight patients were successfully weaned and discharged from hospital. One patient died on ECMO due to cerebral bleeding and one patient died 8 days after weaning due to septic circulatory failure. The control group consisted of 13 (6 male, 7 female) healthy volunteers with a median age of 24.3 years (23.9/24.4).

**Table 2:** ECMO run data from all patients (n=10) and from the two NET-rate-derived subgroups [HIGH-NET (n=4) and LOW-NET (n=6)] prior to the beginning of ECMO therapy.

	All	HIGH-NET	LOW-NET	p-value
**Patients (n)**	10	4	6	
**Total ECMO-support (days) (median/IQR)**	12 (8/17)	7 (5/102)	15 (9/17)	0.286
Anticoagulation during ECMO therapy				
Anticoagulation with Heparin (n, %) Anticoagulation with Argatroban (n, %)	5, 50 5, 50	2, 50 2, 50	3, 50 3, 50	1.000 1.000
Type of primary ECMO systems				
DP3 system (Fresenius Medical Care) (n, %) Cardiohelp HLS (Maquet) (n, %) PLS (Maquet) (n, %) Life-Box (Liva Nova) (n, %)	3, 30 3, 30 1, 10 3, 30	3, 75 0, 0 1, 25 0, 0	0, 0 3, 50 0, 0 3, 50	
**Survival to hospital discharge (n, %)** Deceased during ECMO* (n, %) Deceased after ECMO** (n, %)	8, 80 1, 10 1, 10	4, 100 0, 0 0, 0	4, 67 1, 17 1, 17	0.467
**Patients with MO-/System exchange (n, %)** 1 MO-/System exchange (n, %) 2 MO-/System exchanges (n, %) 11 MO-/System exchanges (n, %)	4, 40 2, 20 1, 10 1, 10	1, 25 0, 0 0, 0 1, 25	3, 50 2, 33 1, 17 0, 0	0.571
Cause for all MO-/System exchanges*** (n) Device-induced coagulation disorder (n, %) Acute pump head thrombosis (PHT) (n, %) Acute oxygenator thrombosis (AOT) (n, %) Worsening of gas exchange capability (n, %) Unknown (n, %)	15 6, 40 4, 27 1, 7 3, 20 1, 7	11 4, 36 3, 27 1, 9 2, 18 1, 9	4 2, 50 1, 25 0, 0 1, 25 0, 0	

Data are presented as absolute quantities n with proportions or as median (interquartile range). *Cause of death during ECMO: intracerebral hemorrhage; **Cause of death after ECMO: septic circulatory failure; ***Reasons for oxygenator change according to Lubnow et al., 2014 (6); In retrospective analysis, not all reasons for a change of oxygenator could be ascertained without any doubt.

To classify patients according to their NET rate before the initiation of therapy, a cutoff value for group assignment was calculated from the NET rates of the healthy controls using the 3-sigma rule (mean + 3 x standard deviation; NET rate = 1.5% + 5.1% = 6.6%). Four patients (40%) presented blood smears with a NET rate >6.6% and were thus classified as HIGH-NET, the other six patients (60%) were classified as LOW-NET. There were no significant differences in baseline characteristics (except for TNF-α and IL-6) (**Table 1**) and ECMO run data (**Table 2**) between the HIGH-NET and LOW-NET groups.

### 3.3 NETs and their precursors in the bloodstreams of critically ill patients prior to ECMO therapy

**Figure 2:**
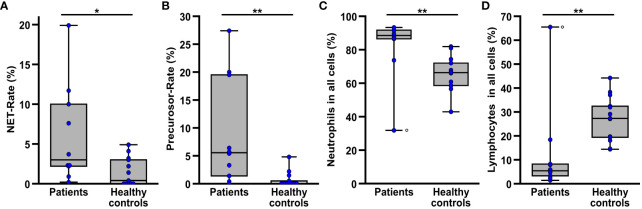
Comparison of NET rate **(A)**, NET-precursor rate **(B)** as well as the proportion of healthy neutrophil granulocytes **(C)** and lymphocytes **(D)** among all detected leukocytes in blood smears from patients (n=10) prior to the beginning of ECMO therapy and healthy controls (n=13). The circle (o) marks the datapoints of one patient suffering from chronic lymphocytic leukemia, resulting in a very high proportion of lymphocytes and a very low proportion of neutrophils in the blood smears. *p<0.05 and **p<0.01.

Comparing the blood smears of patients (n=10) prior to initiation of ECMO therapy and healthy controls (n=13) (**Figure 2**), the patients showed significantly higher rates of NETs (p=0.018) and their precursors (p=0.001) (**Figures 2A, B**). The proportion of neutrophils in all leukocytes (**Figure 2C**) was also significantly elevated (p=0.002) in the patients, whereas the proportion of lymphocytes (**Figure 2D**) was significantly lower (p=0.002) compared to the healthy controls. The proportion of monocytes and eosinophils in all cells showed no significant differences in the comparison between patients and controls (**Supplementary Figure S7**).

### 3.4 Patients with a high NET rate at initiation of ECMO therapy show higher levels of TNF-α and IL-6

**Figure 3:**
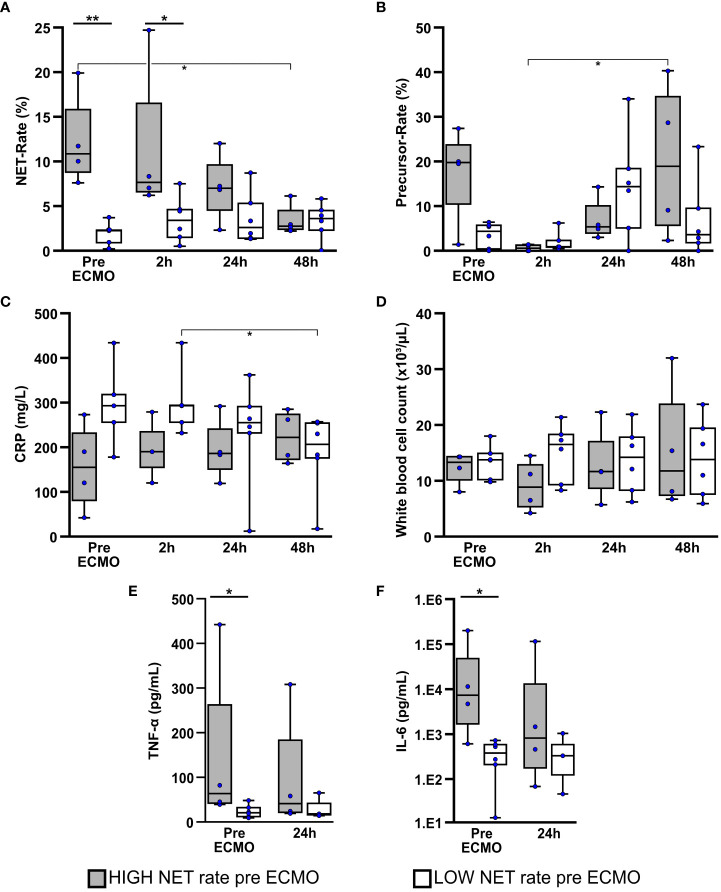
Patients with a high NET rate at initiation of ECMO therapy presented higher levels of TNF-alpha and IL-6. Comparison of NET- **(A)** and NET-precursor rates **(B)** in bloodsmears as well as biomarkers for infection and inflammation which included CRP **(C)**, white blood cell count **(D)**, TNF-α **(E)** and interleukin-6 (IL-6) **(F)** over the first 48h of ECMO therapy in Patients with high or low NET-rate at the time of ECMO initiation. *p<0.05 and **p<0.01.

The first 48h of ECMO therapy were evaluated regarding NET- (**Figure 3A**) and precursor rates (**Figure 3B**) in blood smears and several markers of inflammation and infection (**Figures 3C–F**) considering the NET rate derived subgroups.

Blood smears of the HIGH-NET group presented a significantly higher NET rate (**Figure 3A**) before (p=0.005) and 2h after (p=0.038) initiation of ECMO therapy compared to the LOW-NET group. Over the first 48h after therapy initiation, the NET rate showed a significant decrease (Friedman: p=0.012; adj. p_(48h-pre ECMO)_=0.016) with a reduction in the median NET rate of approximately 75% [median pre ECMO: 10.9% (IQR 8.8%/15.8%); median after 48h: 2.8% (IQR 2.4%/4.5%)]. The NET rate in the LOW-NET group, however showed no significant changes over that time, therefore a convergence of the NET rate of both groups could be observed.

Compared to the LOW-NET group, the HIGH-NET group presented initially an increased precursor rate (**Figure 3B**), but without reaching significance (p=0.114). 2h after initiation of ECMO therapy, the precursor rate showed a significant minimum in patients in the HIGH-NET group (Friedman: p=0.019; adj. p_(48h-2h)_=0.037). No significant changes were observed in the LOW-NET group.

CRP levels (**Figure 3C**) presented slightly but not significantly increased in the blood samples of the LOW-NET group before (p=0.111) and 2h after (p=0.143) initiation of ECMO therapy compared to the HIGH-NET group. Over the first 48h of treatment, the CRP levels in the LOW-NET group showed a significant decrease (Friedman: p=0.007; adj. p_(48h-2h)_=0.042) whereas the HIGH-NET subgroup presented no significant changes. White blood cell counts (**Figure 3D**) did neither show significant differences in the comparison of the two groups, nor in the respective time course over 48h.

Prior to ECMO therapy, patients assigned to the HIGH-NET group showed significantly higher levels of both TNF-α (p=0.038; **Figure 3E**) and IL-6 (p=0.019; **Figure 3F**) as biomarkers for inflammation compared to the LOW-NET group. Over the first 24h of therapy, a convergence of this difference in biomarker levels was observed. A Spearman rank correlation analysis across all patients showed a significant correlation between TNF-α and IL-6 and both NETs (IL-6: p=0.003, rho=0.827; TNF-α: p=0.001, rho=0.863) as well as their precursors (IL-6: p<0.001, rho=0.879; TNF-α: p<0.001, rho=0.927). Corresponding scatterplots can be found in the (**Supplementary Figure S8**).

The comparative analysis of neutrophils, lymphocytes, monocytes and eosinophils in the blood smears (**Supplementary Figure S9**) over the first 48h of ECMO therapy showed largely no significant differences between the two groups except for a significant increase in detected monocytes in the HIGH-NET group 24h after the start of therapy (Friedman: p<0.001; adj. p_(24h-pre ECMO)_=0.042), which could not be observed in the LOW-NET group resulting in a significant difference between both groups (p=0.010).

### 3.5 Patients with a high NET rate at the time of ECMO administration show a greater decrease in platelet counts and ATIII activity

To assess acquired coagulation disorders during ECMO therapy, platelet counts, parameters of plasmatic coagulation, along with the presence of disseminated intravascular coagulation (DIC) were analysed over the first 48h of therapy.

**Figure 4:**
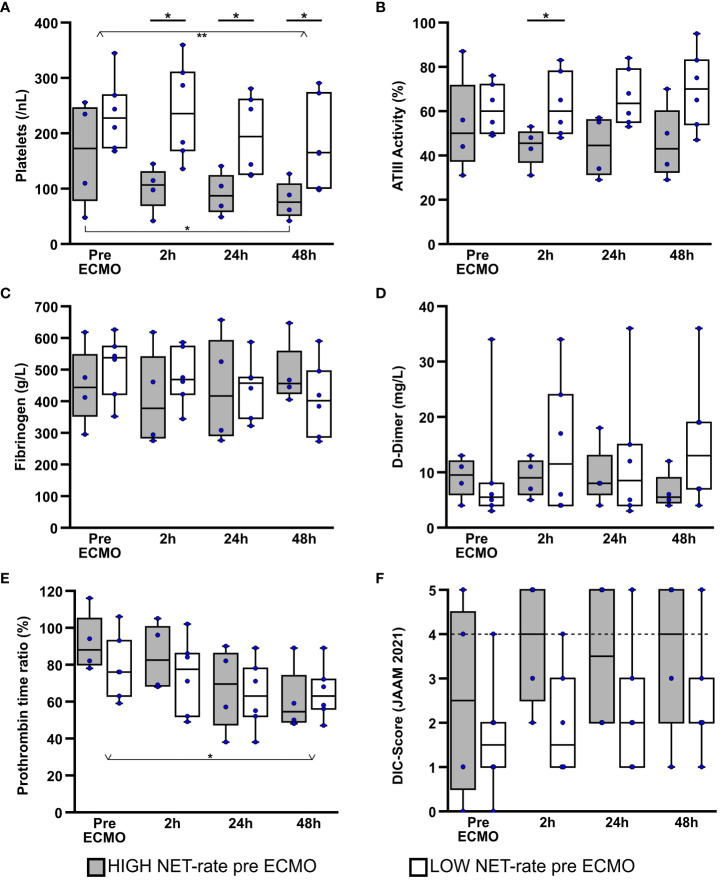
Comparison of laboratory data regarding coagulation over the first 48h of ECMO therapy. Platelet counts **(A)**, antithrombin III (ATIII) activity **(B)**, fibrinogen **(C)**, D-dimer **(D)**, and prothrombin time ratio **(E)** were examined, taking into account patient groups defined by NET rate. Disseminated intravascular coagulation (DIC) was predicted according to the revised Japanese Association for Acute Medicine (JAAM) diagnostic criteria (50) **(F)**.The dashed line symbolizes the cutoff point of 4 at which the 2016 JAAM DIC diagnostic criteria is considered positive for the presence of disseminated intravascular coagulation. *p<0.05 and **p<0.01.

The combined collective of all patients (Friedman: p=0.001; adj. p_(48h-pre ECMO)_=0.003), as well as the HIGH-NET subgroup (Friedman: p=0.014; adj. p_(48h-pre ECMO)_=0.024) showed a significant decrease of platelets after initiation of ECMO therapy (**Figure 4A**). The LOW-NET group showed a similar trend, but without reaching significance (Friedman: p=0.016; adj. p_(24h-pre ECMO)_=0.003). However, the effect could be observed earlier and was particularly strong in the HIGH-NET group [median pre ECMO: 173/nL (IQR 79/246); median after 48h: 76/nL (IQR 52/108)], whereas the LOW-NET group showed a less prominent and also delayed decrease in platelet counts [median pre ECMO: 228/nL (IQR 174/269); median after 48h: 165/nL (IQR 101/273)]. This was also reflected in the direct comparison of platelet counts between both subgroups. Before ECMO initiation, there was no significant difference between both groups, but this difference could be observed already 2h after therapy initiation (p=0.019) and further on during the first 48h of therapy (p=0.038 after 24h and 48h).

When looking at antithrombin III activity (ATIII) (**Figure 4B**), there was a slight, although not significant decrease in the HIGH-NET group after the start of ECMO therapy [median pre ECMO: 50.0% (IQR 37.5%/71.5%); median after 48h: 43.0% (IQR 32.5%/60.0%)]. In contrast, patients in the LOW-NET group presented a slight, also non-significant, increase in ATIII activity over the first 48h of therapy [median pre ECMO: 60.0% (IQR 50.0%/72.0%); median after 48h: 70.0% (IQR 54.0%/83.0%)]. After there was no difference in ATIII activity between the two groups before the start of therapy, a significant difference (p=0.043) was observed after 2h. After 24h (p=0.076) as well as after 48h (p=0.114) there was still a tendency, but without reaching statistical significance.

Evaluation of fibrinogen (**Figure 4C**) and D-dimers (**Figure 4D**) did not reveal any differences, neither in the comparison between the two subgroups nor in the time course over the first 48h of ECMO therapy.

In the analysis of the prothrombin time ratio (PR) (**Figure 4E**), a significant decrease (Friedman: p=0.011; adj. p_(48h-pre ECMO)_=0.044) of the PR in the course of therapy [median pre ECMO: 80.0% (IQR 76.0%/94.0%); median after 48h: 58.5% (IQR 50.0%/72.0%)] was observed when all patients were considered together. In the subgroup analysis this decrease could only be recognized as a tendency (HIGH-NET Friedman: p=0.052; LOW-NET Friedman: p=0.151), but without reaching significance. When a direct comparison between the two groups was made, no statistically significant differences were found at any of the time points examined.

Including platelet counts, international normalized ratio (INR) derived from prothrombin time rate, D-dimers as fibrin cleavage products and antithrombin III activity, disseminated intravascular coagulation (DIC) was predicted according to the revised Japanese Association for Acute Medicine (JAAM) diagnostic criteria (50) (**Figure 4F**). A score value ≥4 was considered to be indicative for the presence of DIC. During the first 48h of ECMO treatment, 50% (n=2) of patients in the HIGH-NET group showed a DIC score ≥ 4 at each measurement time point. In the LOW-NET group, only 17% (n=1) of patients showed increased score values to this extent. Direct comparison of the two subgroups showed a trend towards higher scores in the HIGH-NET group, but no significant differences were observed.

Free hemoglobin and lactate dehydrogenase (LDH) were examined as indicators for hemolysis (**Supplementary Figure S10**). No significant differences were observed either in the comparison between the subgroups or in the time course over the first 48h of ECMO therapy.

Due to random selection of our patients half of the patients were anticoagulated with heparin (n=5) and argatroban (n=5). Both anticoagulation regimens were compared regarding NET and precursor rate, as well as coagulation parameters (**Supplementary Figures S11, S12**). There were no significant differences in the NET or precursor rate. Patients treated with heparin showed slightly lower platelet counts after 24h and 48h but without reaching significance (p=0.222). All other coagulation parameters as well as the calculated DIC score were comparable.

### 3.6 System exchanges due to complications

**Figure 5:**
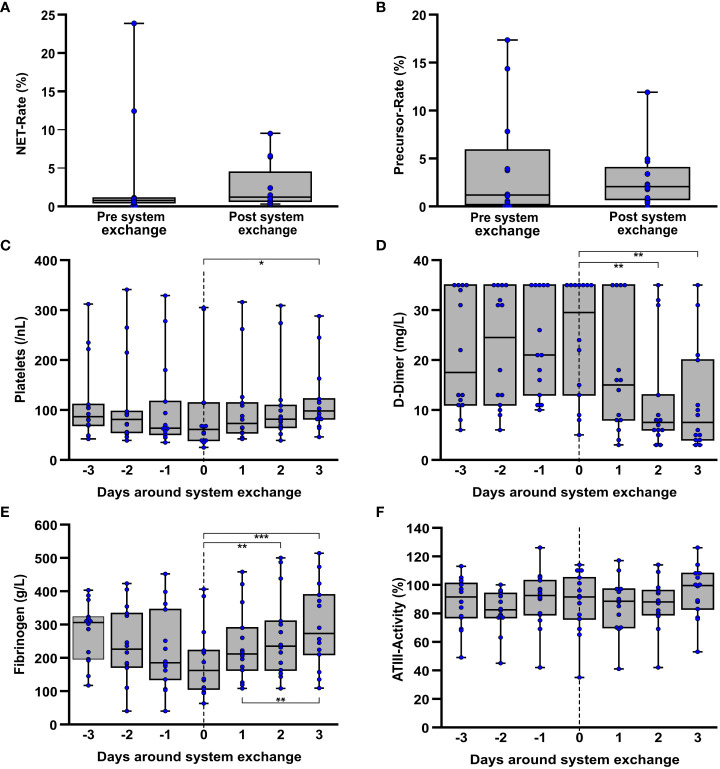
Laboratory data on coagulation in the interval around ECMO system replacement due to an ECMO-associated complication. If they were obtained around an exchange (n=12) the NET- **(A)** and precursor rates **(B)** each contain the last measured value before and the first value after a system exchange, regardless of how many days lay between the time points. Furthermore, platelet counts **(C)**, D-dimers **(D)**, fibrinogen **(E)**, and ATIII activity **(F)** were considered over the period around a system exchange. Here, each of the exact time points of almost all individual changes (n=14) were included. *p<0.05, **p<0.01 and ***p<0.001.

Due to the overall small sample size with only 4 patients requiring a system exchange and the rapidly diminishing differences between the HIGH-NET and LOW-NET groups within the first 48h of therapy, no further subgroup analysis was performed and all respective patients were considered together (**Figure 5**). Blood smears before and after the exchange could be obtained for 12 of the total 15 system exchanges. Data from 9 of these exchanges originated from a single patient with an exceptionally long course of ECMO-therapy. When comparing NET (**Figure 5A**) and precursor rates (**Figure 5B**) in the blood smears before and after the exchange events, no significant differences were found.

With exception of a single event due to the patient’s death, all system exchanges could be studied together within a 3-day interval before and after. In this regard, a significant (Friedman: p=0.045; adj. p_(3d post exchange-exchange day)_=0.041) increase in platelet counts (**Figure 5C**) was observed following a system exchange [median day of exchange: 61/nL (IQR 39/114); median 3d post exchange: 98/nL (IQR 82/122)]. A decreasing tendency (Friedman: p=0.097) was seen prior to the exchange but without reaching significance. However, in a similar manner a highly significant (Friedman: p<0.001; adj. p_(3d post exchange-exchange day_<0.001) increase in fibrinogen concentration was observed [median day of exchange: 162g/L (IQR 106/222); median 3d post exchange: 273g/L (IQR 210/389)], following a non-significant (Friedman: p=0.079) decrease (**Figure 5E**) prior to the system exchange. D-dimers (**Figure 5D**) also showed a highly significant (Friedman: p<0.001; adj. p_(3d post exchange-exchange day_=0.001) decrease following the system exchange whereas no significant differences in ATIII activity (**Figure 5F**) could be seen during the period around a system exchange. Both LDH (Friedman: p=0.011; adj. p_(2d post exchange-exchange day_=0.020) as well as free hemoglobin (Friedman: p=0.050) as indicators of hemolysis showed a decrease following the exchange. The corresponding graph is displayed in the supplement (**Supplementary Figure S13**).

Further operational parameters of ECMO therapy around system exchanges concerning blood- and gasflow as well as partial pressures of CO_2_ and O_2_ in the blood at the outlet of the membrane oxygenator are presented in **Supplementary Figure S14**. In this regard, a reduction in ECMO gas flow (Friedman: p=0.031) could be observed following the system exchange. ECMO blood flow und post-oxygenator pO_2_ showed only trends but no significant differences. Post-oxygenator pCO2 also showed no significant differences.

In order to evaluate the influence of the significantly overrepresented patient in the pooled analysis, a subsidiary investigation considering only the first exchange of each patient (n=4) was added (**Supplementary Figure S15**). These results were mostly consistent, in particular there were also no differences in the NET and precursor rate before and after the exchange. The trend in fibrinogen and D-dimer levels could be observed to the same extent as in the pooled evaluation. Contrary to the prior observation, platelet counts did not show any significant changes, which, however, might be explained by the small sample size with relatively large dispersion.

## 4 Discussion

To the best of our knowledge, this study is the first to identify NETs and NET precursors in blood smears of patients receiving VV ECMO therapy by using an all-encompassing algorithm based on immunofluorescence microscopy.

When reviewing different methods for the detection and quantification of NETosis, the preparation of blood smears with subsequent fluorescence staining and digital quantification turned out to be a promising approach due to the comparatively simple and well reproducible methodology. NETs have already been detected in blood smears by using fluorescence microscopy (31, 32) and approaches for digital NET quantification have been described before (28, 47, 52). We also aimed to evaluate the amount of NET precursors in the blood smears, as these have been widely described (27, 29, 30) but not adequately detected and quantified in this context.

In our study, in addition to shape and size descriptors, we used DAPI as a DNA-binding dye as well as specific antibodies against CD66b (32, 42, 53, 54) and MPO (45, 46, 48) to reliably identify all individual DNA containing entities and to distinguish them from non-blood cell-associated DNA or other artifacts in the blood smear. Other, more NET specific antibodies, such as those against citrullinated H3 or neutrophil elastase (27, 28, 31, 32, 55) were also evaluated and tested, but presented enormous fluctuations regarding intensity and also very selective staining, so that NET filaments in particular could not be identified with the sensitivity and specificity required for this evaluation. This was seen both in the positive controls as well as in the blood smears and would have made the desired automated and thus investigator-independent evaluation, based on fixed criteria almost impossible. Also, these target antigens were incapable of correctly distinguishing the viable nucleated cells in the blood smear, which was certainly possible with MPO and CD66b. The specificity of NET detection with the antibodies used is slightly limited compared to more NET-specific staining. However, we consider the antibodies used to be suitable of realising an automated analysis of all objects in the blood smear with high accuracy.

The acquired fluorescence images of the blood smears were subsequently processed and measured semi-automatically using an ImageJ macro. Categorisation of the detected and measured objects were carried out using a multi-parameter based algorithm. This approach allowed not only to calculate the proportion of NETs and NET precursors, but in addition, statements could be made about the individual subpopulations of nucleated cells, especially neutrophil granulocytes and lymphocytes, as major players in infection and inflammation. By using multiple parameters to define the different cell nuclei, NETs and NET precursors, the algorithm addressed the challenge that both cell nuclei and NET filaments in blood smears can widely vary in appearance and are therefore sometimes difficult to distinguish based on a single parameter. However, this described variability could not be fully compensated by a rigid algorithm, which inevitably leads to limitations in terms of sensitivity and specificity in the detection and classification of the individual objects in the blood smears. In this regard, a future optimization of the evaluation by integration of intelligent deep-learning algorithms or a complementary NET-detection in blood plasma e.g. by an NET-ELISA (55–57) can be discussed.

Apart from these limitations, the methodology proved to be suitable for differentiation and quantification of NETs, their precursors as well as all other DNA-containing objects in blood smears. In particular, the comparability between the individual blood smears is given without restriction due to the uniform and user-independent evaluation, as well.

Further, unavoidable limitations result from the choice of blood smears as the analyzed material and are due to various possible interfering factors during sample collection and subsequent processing. Parts of the thin and therefore very fragile NET filaments may be lost or destroyed due to shear stress of blood collection, pipetting and smearing, which was reflected in a tendency towards atypical NET forms and especially shorter, thicker NET filaments to be detected in the blood smears. On the other hand, NET formation may also be induced by high shear forces (40) (e.g. during blood collection or subsequent transport) or due to exposure to circulating, NET inducing cytokines present in the blood.

As expected, the blood smears from critically ill patients that required VV ECMO (n=10) showed significantly increased rates of NETs and NET precursors as well as a higher proportion of neutrophils in all nucleated cells compared to healthy controls (n=13) as a correlate of the severe generalized inflammation. Blood samples of the same patient population also contained significantly higher plasma levels of cell free DNA (cfDNA) compared to healthy controls (58). The results of our present study are consistent with a comparable immunofluorescence microscopic analysis of blood smears from critically ill patients with systemic inflammatory response syndrome (SIRS) and healthy volunteers, in which NETs were detected frequently in the patient’s blood (31, 32). NETs could also be detected in the blood of patients with infectious acute respiratory distress syndrome (ARDS) (56), in transfusion-related acute lung injury (57) and were associated with a more severe lung injury and a higher mortality (56). The fact that NETs could also be detected to a certain extent in the healthy subjects may be partly interpreted as a consequence of the aforementioned NET induction by blood collection as well as sample transport and processing, since we also simulated these in the subject collective to ensure equal treatment of the samples. However, the blood of the subjects contained hardly any NET precursors, which we attributed to the merely selective activation of individual granulocytes. In contrast, large amounts of NET precursors with decondensed nuclei were found in the largely septic patient population, presumably due to ubiquitous inflammation with extensive neutrophil activation and NET induction.

To improve the correlation of inflammation and coagulation biomarkers and clinical outcome with the NET rate, the patient population was divided into a “HIGH-NET” and a “LOW-NET” group based on the NET rate before initiation of ECMO therapy, as described previously (59, 60). Basic patient characteristics such as gender, age and BMI showed no significant differences between both groups. The leading cause of lung failure in the HIGH-NET group was bacterial pneumonia with 50%, whereas viral pneumonia was more likely to be the cause in the LOW-NET group with 67%. This observation, although not statistically significant due to the small patient population, is consistent with the widely described experience that bacteria and bacterial proteins, in particular, are potent NET inductors (21, 27, 29). Although SOFA score and lung injury score did not show significant differences, patients in the HIGH-NET group tended to be ventilated longer before initiation of ECMO therapy. However, the median duration of ECMO support was longer in the LOW-NET group, although also not significant.

When comparing inflammatory parameters, patients of the HIGH-NET group presented significantly increased TNF-α and IL-6 levels compared to patients with a low NET rate, whereas CRP and WBC showed no significant differences. The synergistic and reinforcing relationship between NET formation and the release of proinflammatory cytokines and chemokines, such as TNF-α, IFN-α, IL-6, IL-18, and IL-8 with a consecutive enhancement of the immune response well as the formation of NETs has been described frequently (33–35, 61, 62) and also our data showed a highly significant correlation between the NET-inducing cytokines TNF-α and IL-6 both with the detection of NETs as well as their precursors in the blood before the start of ECMO therapy. A self-sustaining vicious circle of NET formation and cytokine release has already been identified as an important pathophysiological factor in chronic inflammatory processes (55, 61) and in acute infection or SIRS the excessive cytokine release represents an important factor of mortality (62–64).

A marked decrease in cytokine levels already 24h after initiation of ECMO therapy described by Burell et al. (65) was also observed in this study, especially in the patients with a high NET rate and was accompanied by a significant decrease of the NET rate. This led to a rapid convergence of the initially large differences in the NET rate between the two groups, so that already after 48h no significant difference was detectable. Besides the drop in cytokine levels after initiation of ECMO therapy, adhesion of neutrophils, NETs and their precursors on the surface of the large gas exchange membranes or other artificial surfaces might also provide a partial explanation for the decrease and would be consistent with previous observations (19, 39, 41, 66).

Additional neutrophil activation due to exposure to circulating cytokines could play a role at the time of therapy initiation as these blood samples were only processed together with the 2h samples as a result of the transport of the patient to the ICU from external hospitals. However, this delay was of comparable duration in both groups and the 2h samples still showed a significant difference in NETs between the two groups, which makes this delay as the leading cause of difference improbable. Furthermore, a time period of 2-3h is rather at the minimum time required for NET formation (27, 28, 30), so that only few additional NETs would be expected. But as the process of nuclear decondensation begins shortly after stimulation (27, 28, 30), an increased occurrence of NET precursors in the samples with a high cytokine burden before the start of ECMO is plausible and expected. This might also provide further explanation for the initial high amount of NET precursors followed by an absolute minimum after 2h in the HIGH NET group, which could not be observed to the same extent in the LOW-NET group with lower cytokine levels.

The gradual increase in the precursor rate after the start of ECMO therapy may be considered as a result of renewed NET induction. Since this increase occurs to the same extent in both subgroups and is also independent of the initial cytokine levels, this appears to be a therapy-associated effect, e.g. due to artificial surfaces, high shear forces in the oxygenator, etc. These considerations are supported by the results of a study by Zhang et al. (67), which showed an increase in cfDNA and increased NET formation of isolated granulocytes following ECMO treatment over 6h in an animal model. The fact that the NET filaments detected in the blood smears do not increase to the same extent as their precursors may actually be associated with a filter effect of the membrane oxygenator or the disintegration of the long, thin and therefore fragile NET filaments through high shear forces in the ECMO system.

There are countless references in the literature for the thrombogenic potential of NETs in the context of infections (25, 26, 68) and with coagulation-associated technical complications, such as clot formation or critical bleeding as a major cause of mortality and morbidity during ECMO therapy (3, 6, 7), a major focus of this study was to examine platelet counts and parameters of plasmatic coagulation during the course of therapy. As frequently described (6, 7, 9, 13, 69, 70), also in this evaluation the collective of all patients, as well as the HIGH- and the LOW-NET subgroups showed a significant decrease in platelet counts after ECMO initiation. It was noticeable that patients with a high NET rate prior to ECMO initiation presented a more pronounced and earlier platelet drop, as well as a significantly reduced antithrombin III activity immediately after initiation of therapy compared to the patients with an initial lower NET rate. Fibrinogen and D-dimers showed no significant differences between the two groups in this study, nor did they change significantly during the initial 48h of therapy. The anticoagulation used had no influence on NET or precursor rate and as reported by Fisser et al. (51), the patients receiving heparin showed slightly, albeit not significantly, lower platelet counts after 24h and 48h. However, the anticoagulation used was equally distributed among both groups.

Zhang et al. have already shown *in vitro* that thrombin activation of platelets is partly responsible for increased NET formation with expression of tissue factor (TF) in ARDS patients and thus represents a relevant contribution to the development of immunothrombosis (26). By Middelton et al, microthrombi from platelets, NETs, and neutrophils, as well as mutual activation of NETs and platelets, were detected in patients with covid-19 pneumonia (25).

In this context, a self-reinforcing cycle of preexisting inflammation with formation of NETs and activation of coagulation and in particular platelets, as well as an additional amplification of both processes by the proinflammatory (17) and further platelet and coagulation activating effects of ECMO therapy (12, 13) leading to progressive clot forming with an incipient consumption coagulopathy in patients with an initially high NET rate is conceivable here and is partly reflected by the, however, not significantly increased DIC score of these patients.

Technical complications occur in 30-51% of non-COVID-19 and COVID-19 patients during ECMO therapy, leading to an exchange of ECMO components after a median of 9-10 days (6, 7). In this study, technical complications requiring replacement of ECMO components, mostly the membrane oxygenator, also occurred in 4 patients (40%). A total of 15 exchange events occurred, with 11 of these exchanges related to one patient. Under the aspect of a NET-mediated coagulation disorder as a possible cause of technical complications (19, 39, 41), the period around a replacement of parts of the ECMO system should also be investigated with regarding NETosis.

To further investigate technical complications leading to a change of the whole ECMO system or particular components, whenever it was possible, blood smears were obtained on days with a component exchange as well as on the following day. However, due to the setting of ECMO treatment and the clinical procedures in the intensive care unit, major difficulties were encountered in obtaining samples exactly around a component exchange during the study. Since most of the changes were urgent and predictable only in the short term, it was often not possible to obtain a sample immediately before an exchange, which is why the last value prior to a change, regardless of its time interval to the event, had to be used in the evaluation. Due to the described difficulties in sample collection, only 12 of the 15 exchange events could be evaluated.

Comparing all samples before and after the assessed exchange events, the rates of NETs and NET precursors did not show any significant differences. Laboratory data, which were collected daily, showed decrease of platelets and fibrinogen prior to the exchange with a subsequent, significant recovery as described. Also noteworthy was the highly significant decrease in the previously markedly elevated D-dimer after an exchange. These results were not only seen in the pooled analysis of all exchanges, in which a single patient was strongly overrepresented, but also in the isolated analysis of only the first replacement of the ECMO system. The observed alterations of the coagulation parameters are in accordance with previous findings of our group (6, 7). In conclusion, signs of acquired coagulopathy were also evident in the context of technical complications during ECMO therapy, although in this study they could not be related to the presence of NETs or NET precursors.

A general statement on the correlation of the NET rate before therapy initiation with the occurrence of complications during therapy seemed not reasonable due to the small number of patients with complications. Therefore, it must be stated that despite the presence of signs for a NET-associated coagulopathy immediately after initiation of ECMO therapy, the study could not establish a general correlation between the detection of NETs or their precursors in the blood and the occurrence of technical complications during ECMO therapy. However, considering the low number of patients with technical complications as well as the not exactly mapped exchange days and the fact that one patient with 11 exchange events, 9 of which were included in the analysis was strongly overrepresented, the significance of this part of the study is very limited.

In conclusion, neutrophil extracellular traps and their precursors are present in blood samples from patients that require veno-venous ECMO. A high NET rate prior to the initiation of ECMO therapy was associated with a high cytokine burden with increased IL-6 and TNF-α levels as an expression of an inflammation-related NET induction. Following therapy initiation, an earlier and more pronounced drop in platelet counts as well as reduced ATIII activity was observed in patients with a high NET rate, indicating the development of an immune-mediated acquired platelet dysfunction/depletion during ECMO therapy. There was also a cytokine-independent increase in NET precursors after the start of therapy, which can be regarded as a consequence of ECMO-induced NET formation. However, this study protocol was not suitable to establish NETs or their precursors as biomarkers for acquired coagulation disorders or complications during ECMO therapy due to several limitations. In this regard, further investigation with a specific focus on technical complications is required. A higher number of patients as well as the daily preparation of blood smears could increase the significance of these parameters as a biomarker.

## Data availability statement

The raw data supporting the conclusions of this article will be made available by the authors, without undue reservation.

## Ethics statement

The studies involving humans were approved by Ethics Committee of the University of Regensburg (vote no. 16-101-0322). The studies were conducted in accordance with the local legislation and institutional requirements. The participants provided their written informed consent to participate in this study.

## Author contributions

MH: Writing – original draft, Conceptualization, Data curation, Methodology, Software, Visualization, Formal analysis, Resources. MF: Writing – review & editing, Data curation, Resources. AP: Writing – review & editing, Data curation, Resources. TM: Writing – review & editing, Conceptualization, Funding acquisition. MG: Writing – review & editing. ML: Writing – review & editing, Data curation, Resources. LK: Writing – review & editing, Funding acquisition, Conceptualization. KL: Writing – original draft, Writing – review & editing, Conceptualization, Methodology, Supervision, Funding acquisition, Project administration.
