# Supplementary material for: Neutrophil extracellular traps - a potential trigger for the development of thrombocytopenia during extracorporeal membrane oxygenation
Source: Front Immunol. 2024 Feb 21;15:1339235. doi: 10.3389/fimmu.2024.1339235 (PMC10914994; doi:10.3389/fimmu.2024.1339235)
Supplement: Supplementary file 1 [file DataSheet_1.pdf]

Supplementary Material

Neutrophil Extracellular Traps - a potential trigger for the development of thrombocytopenia during Extracorporeal Membrane Oxygenation

Haus et al.

Generation of positive controls for the detection of NETs and NET precursors

To generate positive controls for the detection of neutrophil extracellular traps (NETs), Neutrophil granulocytes were isolated from whole blood (in an EDTA-containing tube) of healthy volunteers by double gradient centrifugation (LeukoSpin and PBMC/LymphoSpin medium; pluriSelect Life Science, Leipzig) according to the manufacturer's protocol. Following isolation, the neutrophils were resuspended in RPMI cell medium containing 0.5% human serum and placed onto a glass slide that had been coated with an aqueous 0.01% poly-L-lysine solution for better adherence. After a 25 min adhesion phase at 37°C, the neutrophils were stimulated with 100nM PMA diluted in RPMI medium with 0.5% human serum for 2-3h in a humid chamber at 37°C. Fixation was done with 4% paraformaldehyde at room temperature. Phosphate-buffered saline (PBS) supplemented with bovine serum albumin was used as a washing buffer. The fixed samples were stored in dark conditions at 4°C until staining.

Both the staining protocol for the blood smears as it is described in the method section, as well as the selection of the antibodies used were developed based on these positive controls. In particular, various other antibodies, including more NET-specific antibodies for example against citrullinated histone H3 (citH3) (see Supplement Figure S1 and S2) were also evaluated.

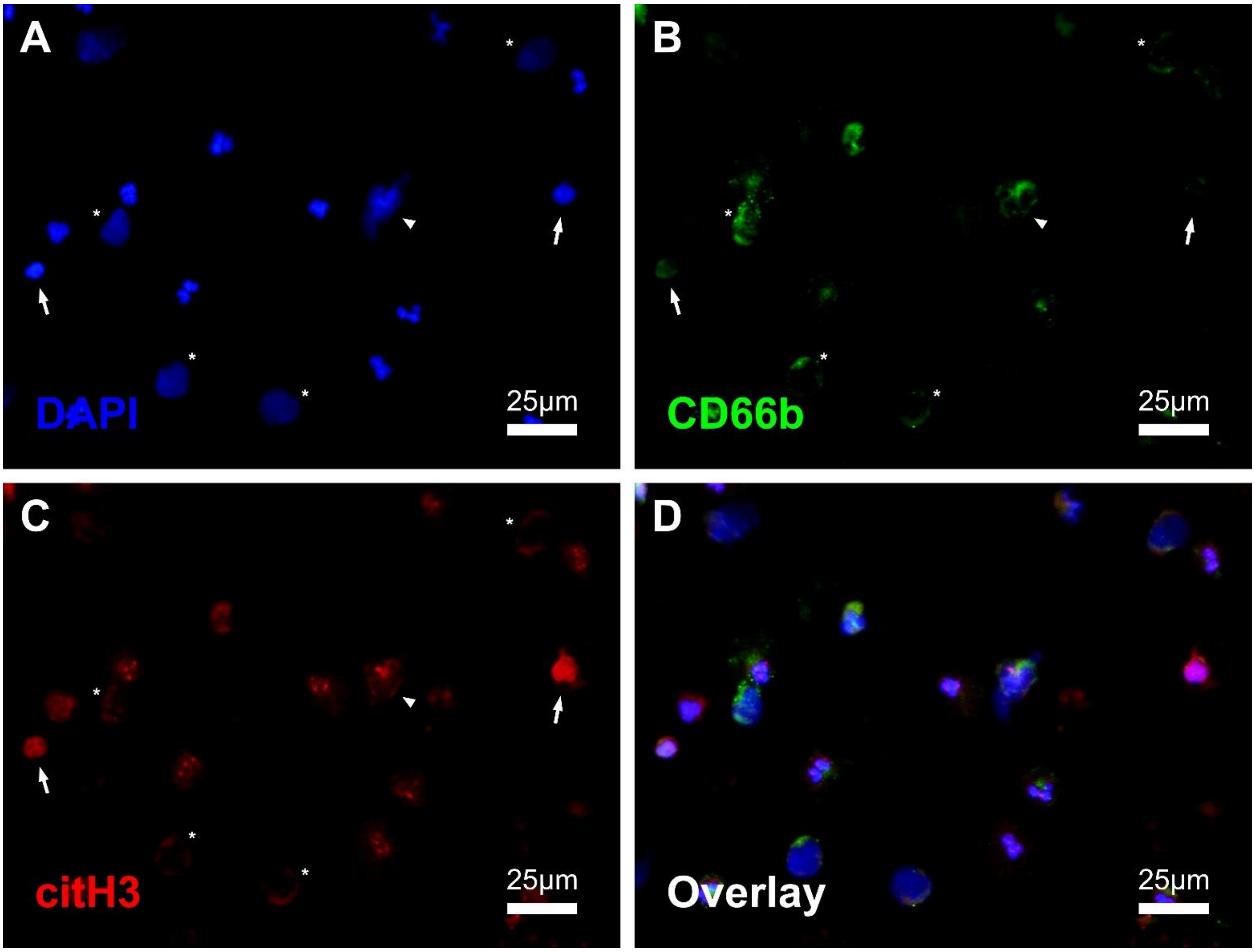

Supplementary Figure S1 Immunofluorescence staining of DNA (DAPI) **(A)**, CD66b **(B)** and citrullinated histone H3 (citH3) **(C)** in NET positive controls, derived from isolated neutrophil granulocytes, stimulated with 100nM PMA for 2h. **(D)** represents the combined overlay. Scale: 25µm

To detect citrullination of histone H3 as a key step in NET formation, a rabbit-derived, antihuman, monoclonal primary antibody against citrullinated histone H3 (citH3) (Abcam, Cambridge, UK; 1:150) was evaluated for a serial analysis besides the primary antibodies ultimately used.

As described in the literature, neutrophils stimulated with PMA for 2h presented a distinct nuclear decondensation (asterisk, *), before NET formation (arrowhead) with expulsion of DNA and granulocytic proteins could be observed. In this context, a high level of citrullinated histone H3 was detected particularly in the small, not yet decondensed cell nuclei (arrow). The larger, already decondensed cell nuclei (asterisk, *), which could also be found in large numbers in some of the blood smears, still showed a clear staining signal, but already a significantly weaker intensity compared to the smaller nuclei. It was also noticeable at this stage that mainly the nuclear edge areas exhibited a positive staining signal. Besides the numerous NET precursors, the beginning of NET formation (arrowhead) could already be found in single cell nuclei after 2 hours.

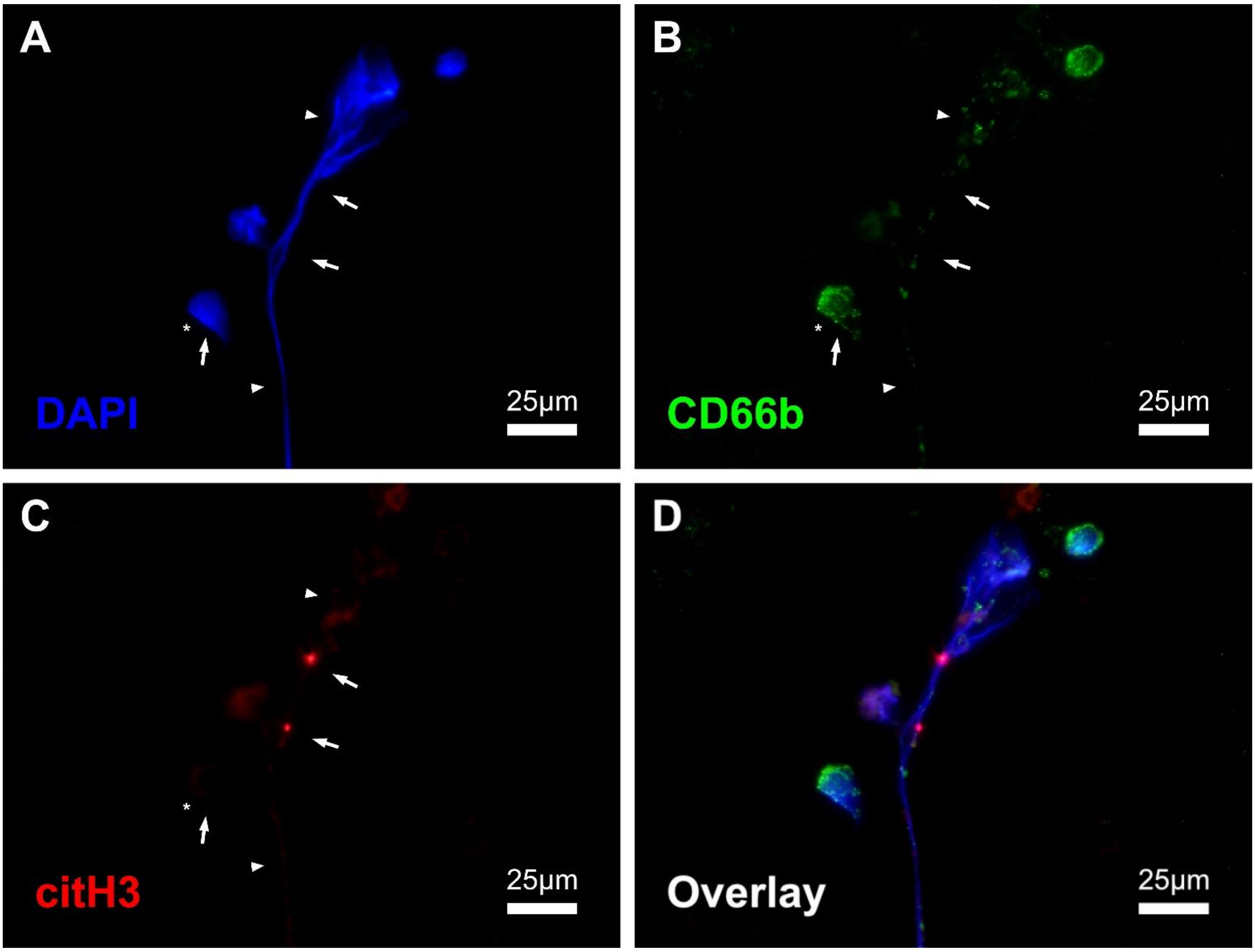

Supplementary Figure S2 Immunofluorescence staining of DNA (DAPI) **(A)**, CD66b **(B)** and citrullinated histone H3 (citH3) **(C)** in NET positive controls, derived from isolated neutrophil granulocytes, stimulated with 100nM PMA for 3h. **(D)** represents the combined overlay. Scale: 25µm

After 3h of PMA stimulation, the typical, string-like NET filaments (arrowhead) as well as smaller, rather atypically configured, maybe earlier NETs (asterisk, *) could be found in large quantities in the positive controls. However, in comparison to the cell nuclei of the early NET precursors, citrullinated H3 could only be detected very selectively/ punctually (arrow) within the individual NET filaments and presented enormous fluctuations regarding signal intensity, which made these antibodies much less favorable for an automated, user-independent evaluation. The antibody against granulocyte marker CD66b showed acceptable results in the detection of neutrophils, NETs and their precursors both in the stimulated granulocytes as well as in the blood smears **(Supplement Figure S6)**.

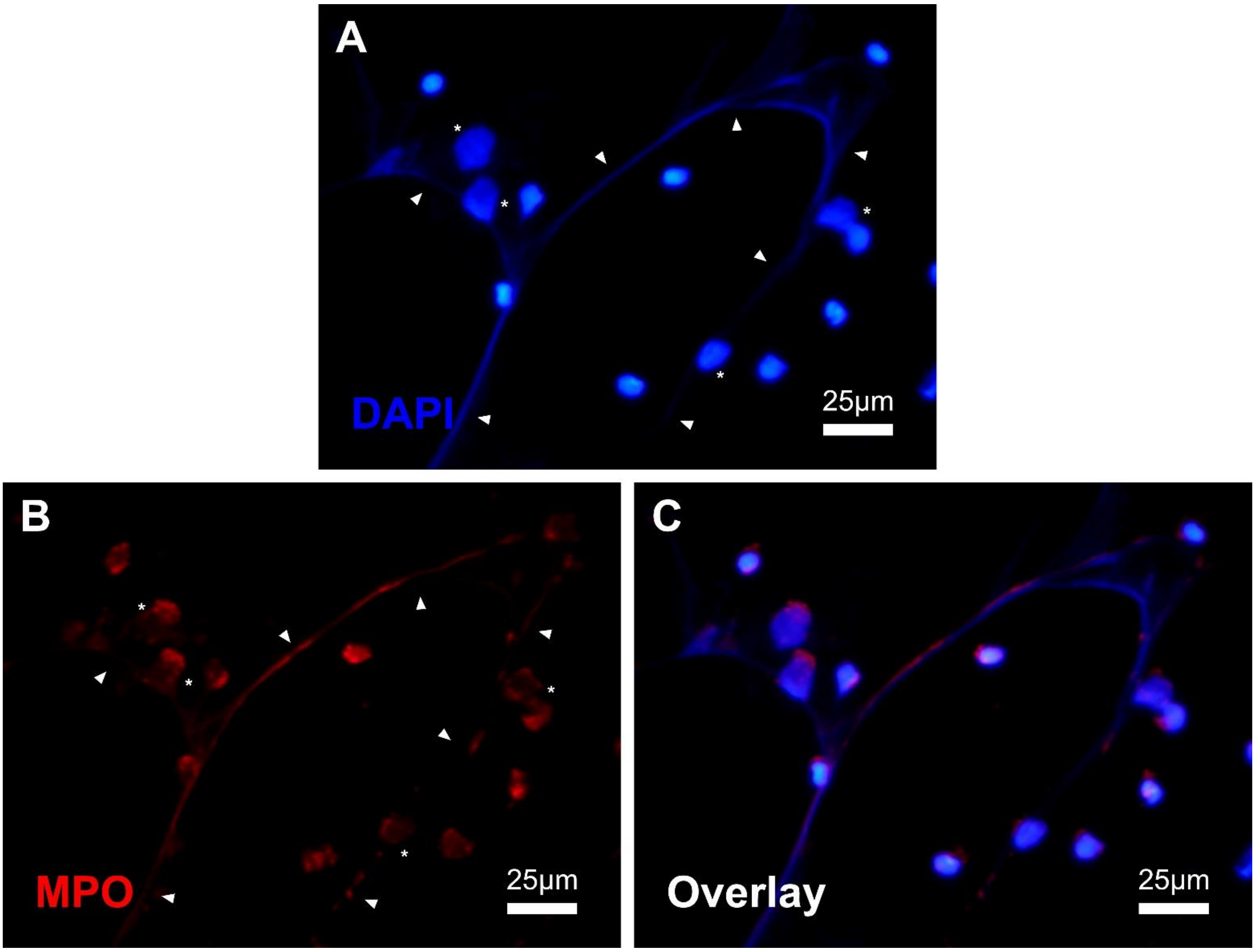

Supplementary Figure S3 Immunofluorescence staining of DNA (DAPI) **(A)** and MPO **(B)** in NET positive controls, derived from isolated neutrophil granulocytes, stimulated with 100nM PMA for 3h. **(C)** represents the combined overlay. Scale: 25µm

In contrast to the antibody staining against citH3 with highly fluctuating intensity and only very selectively detectable signals, staining against MPO yielded very good results, both in the isolated granulocytes and in the blood smears **(Supplement Figure S6)**. In particular, NET filaments (arrowhead) and NET precursors (asterisk, *) showed reliably good staining intensities, which was crucial for automated analysis,

Together with the granulocyte marker CD66b and the various shape descriptors used in the classification algorithm **(Supplement Figure S5)**, the DNA-containing objects in the blood smears could be differentiated reliably.

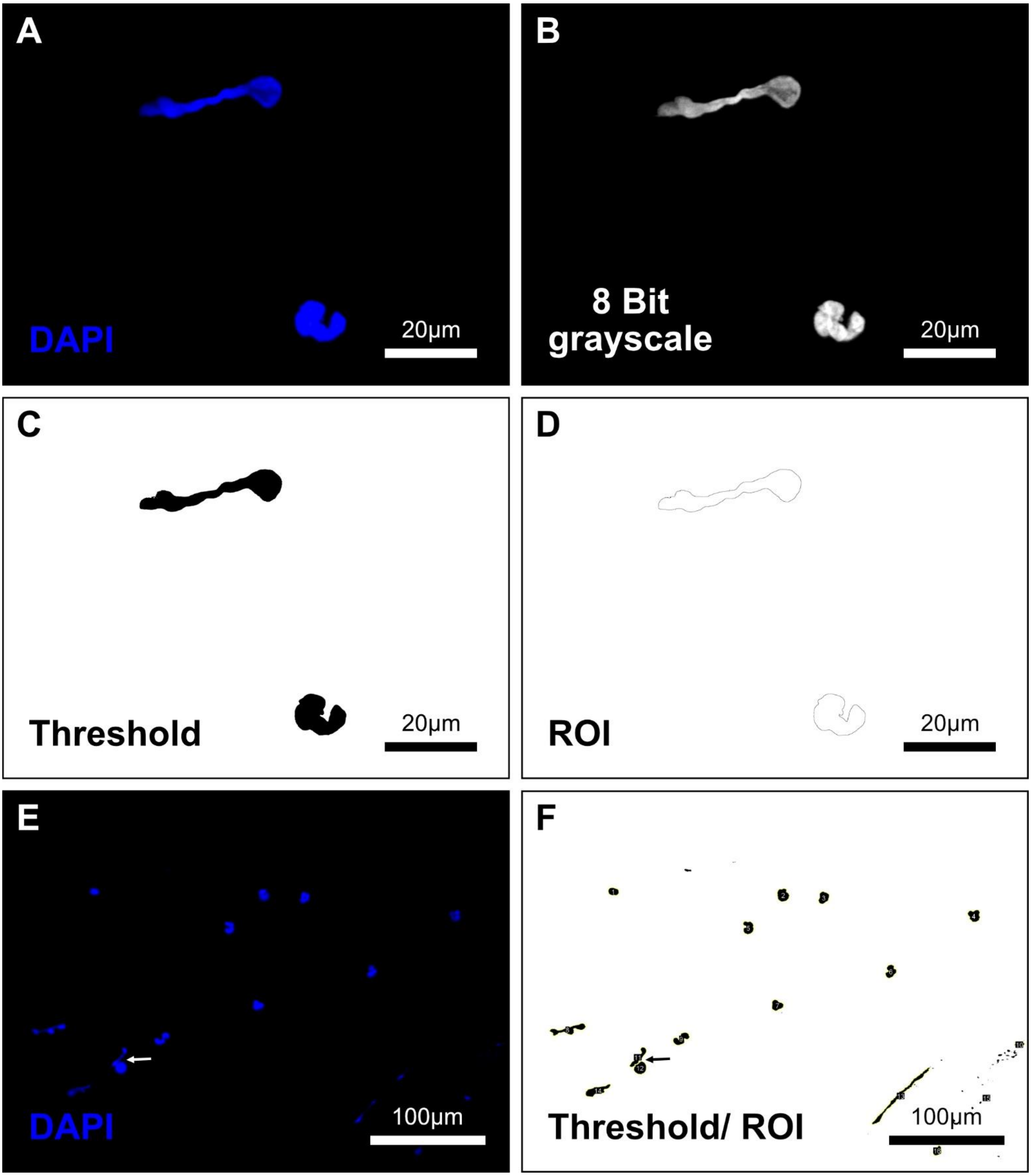

Supplementary Figure S4 Identifying individual objects in fluorescence-stained blood smears using ImageJ. To detect and label each individual object in the blood smear (regular nuclei, NETs, NET precursors, artifacts), the RGB image of the blue DAPI channel for DNA detection **(A, E)** was split into its individual color components (red, green, blue). Initially the 8-bit grayscale image of the blue color component **(B)** was used for the indentification of regions of interest. After adjusting brightness and contrast and adding a filter to sharpen the object outlines, a threshold was applied **(C)** to clearly delineate the objects.

With the generated black and white image, the outlines of all objects with an area ≥ 10µm² were individually stored as region of interest (ROI) in the ImageJ ROI manager **(D, F)**. These ROI templates were then analysed with regard to shape and size and overlayed with the images of each other fluorescent color channel to identify colocalized features. **Figure E and F** show the magnification that was used to analyze all blood smears. When creating the threshold black and white images, there was also the possibility to perform manual corrections in case of obvious errors, such as the overlap of two objects, to avoid a distortion of the results. As an example, the overlap of a regular lymphocyte cell nucleus with a NET filament is shown (**arrow Figure E**), which was corrected manually (**arrow Figure F**). Scale: 20µm (A-D) and 100µm (E-F).

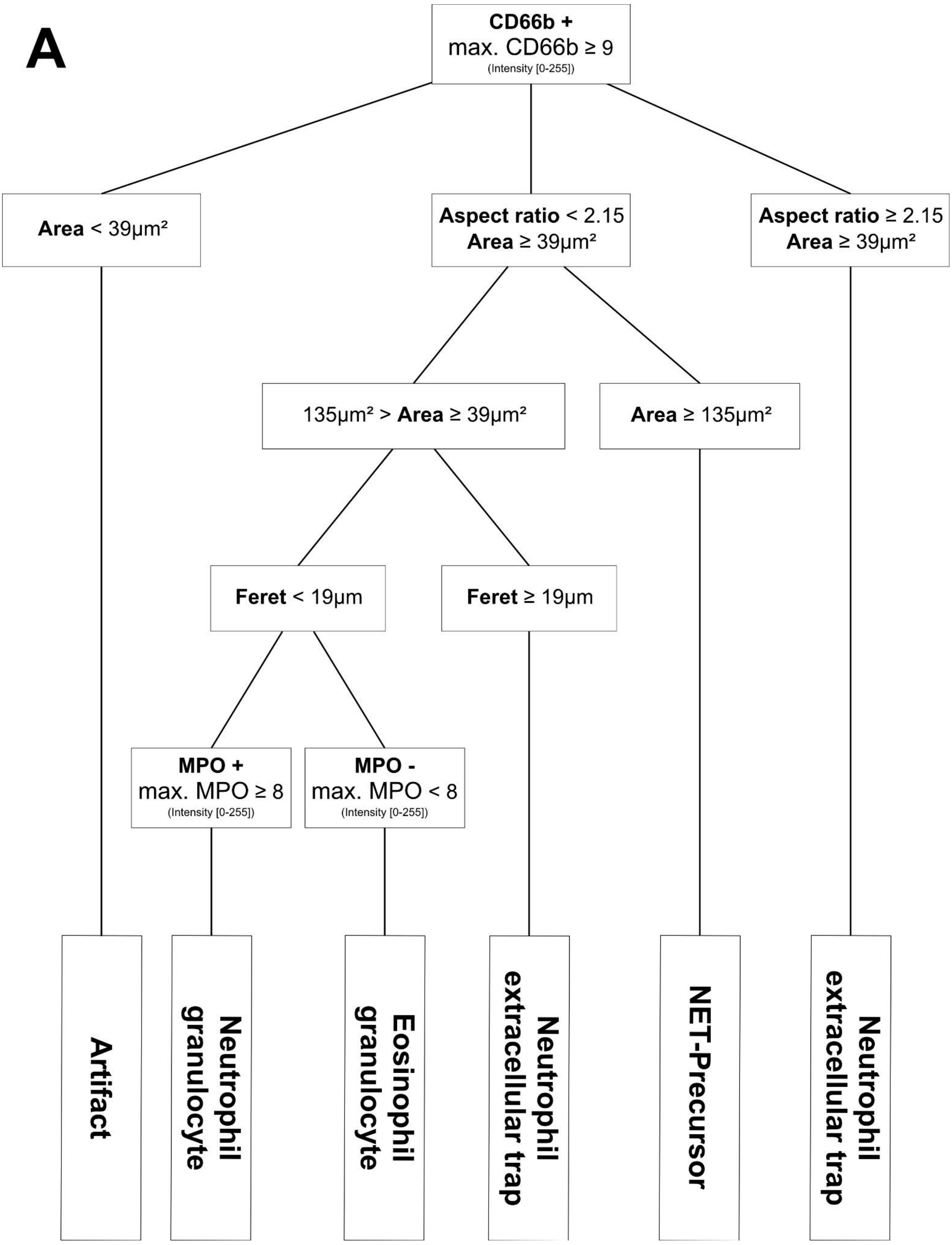
A
CD66b +
max. CD66b ≥ 9
(Intensity [0-255])
Area < 39µm²
Aspect ratio < 2.15
Area ≥ 39µm²
Aspect ratio ≥ 2.15
Area ≥ 39µm²
135µm² > Area ≥ 39µm²
Area ≥ 135µm²
Feret < 19µm
Feret ≥ 19µm
MPO +
max. MPO ≥ 8
(Intensity [0-255])
MPO -
max. MPO < 8
(Intensity [0-255])
Artifact
Neutrophil granulocyte
Eosinophil granulocyte
Neutrophil extracellular trap
NET-Precursor
Neutrophil extracellular trap

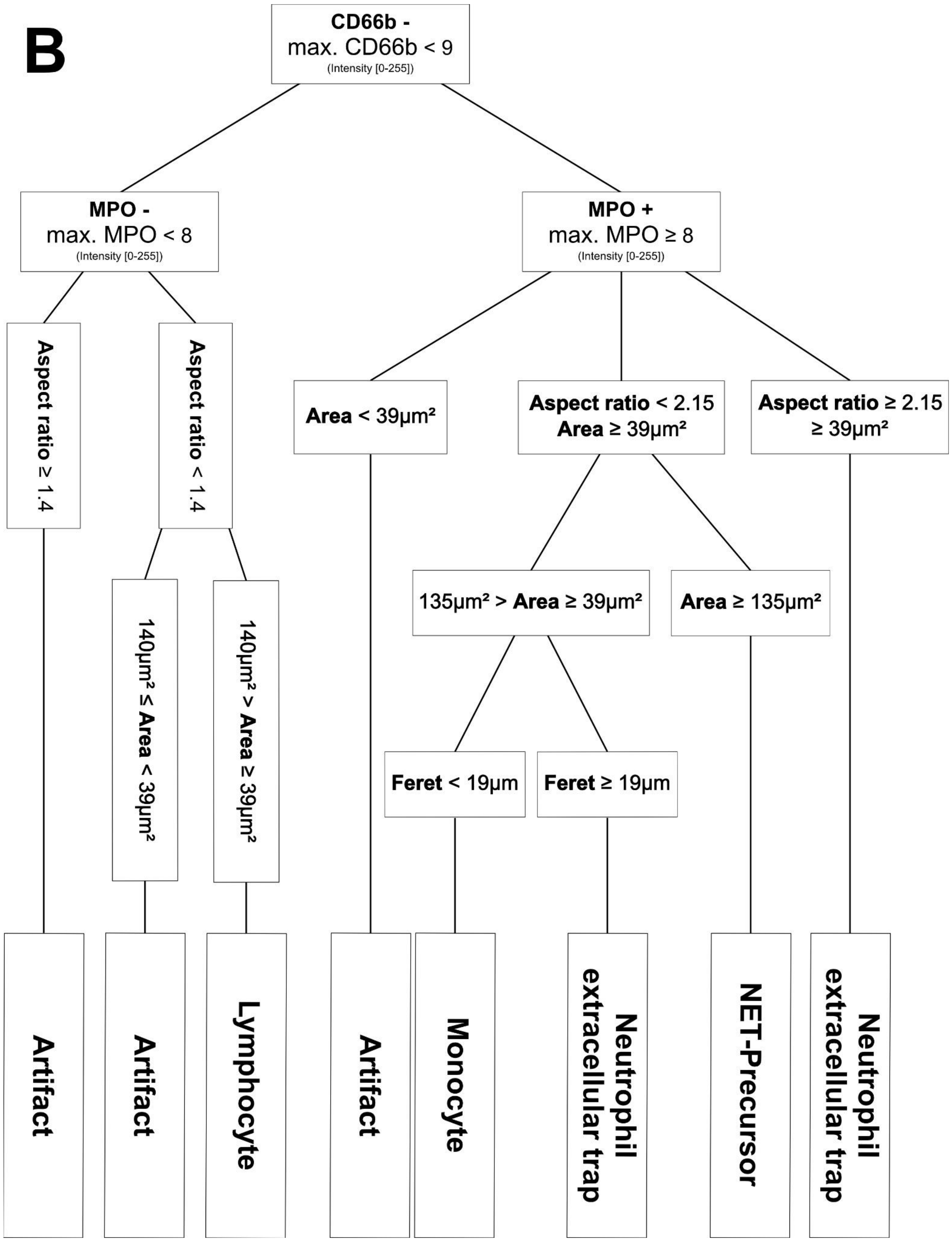
B
CD66b -
max. CD66b < 9
(Intensity [0-255])
MPO -
max. MPO < 8
(Intensity [0-255])
MPO +
max. MPO ≥ 8
(Intensity [0-255])
Aspect ratio ≥ 1.4
Aspect ratio < 1.4
Area < 39µm²
Aspect ratio < 2.15
Area ≥ 39µm²
Aspect ratio ≥ 2.15
≥ 39µm²
140µm² ≤ Area < 39µm²
140µm² > Area ≥ 39µm²
135µm² > Area ≥ 39µm²
Area ≥ 135µm²
Feret < 19µm
Feret ≥ 19µm
Artifact
Artifact
Lymphocyte
Artifact
Monocyte
Neutrophil extracellular trap
NET-Precursor
Neutrophil extracellular trap

Supplementary Figure S5 Categorizing all individual objects identified in the DAPI DNA staining into regular nucleated cells, NETs, NET precursors and artifacts with an automated algorithm. For object differentiation, shape and size of the object outlines identified by DAPI staining as well as the maximum measured intensity (gray level of the 8-bit grayscale image; range 0-255) of the immunofluorescence staining for MPO and CD66b within these obtained object outlines were used. When capturing the microscopic images, we always used the same microscope settings and exposure times. The algorithm consists of two parts **(A, B)**, which are distinguished by the presence of the granulocyte marker CD66b within the outlines of the DAPI stained objects. An object with a maximum gray level ≥9 in the (formerly green) CD66b channel was classified as CD66b positive **(A)** and with a maximum gray level <9 as CD66b negative **(B)**. The detection of MPO within the DAPI stained objects was also based on the maximum gray level of MPO staining. An object with a maximum gray level ≥8 in the red MPO channel was classified as MPO positive, and with a maximum gray level <8 as MPO negative. Further classification was based on the shape and size of the DAPI stained objects. Therefore, the area was used as a general size parameter and the maximum distance between two points on the object's outline ("Maximum Feret's diameter" or "Feret") as well as the ratio of length to width of an ellipse fitted around the object ("Aspect ratio") were used to describe the object's shape. By applying this algorithm to the measured data, specific definitions for all object categories could be established, based on which each DAPI stained object in the blood smear could be reliably assigned to a category.

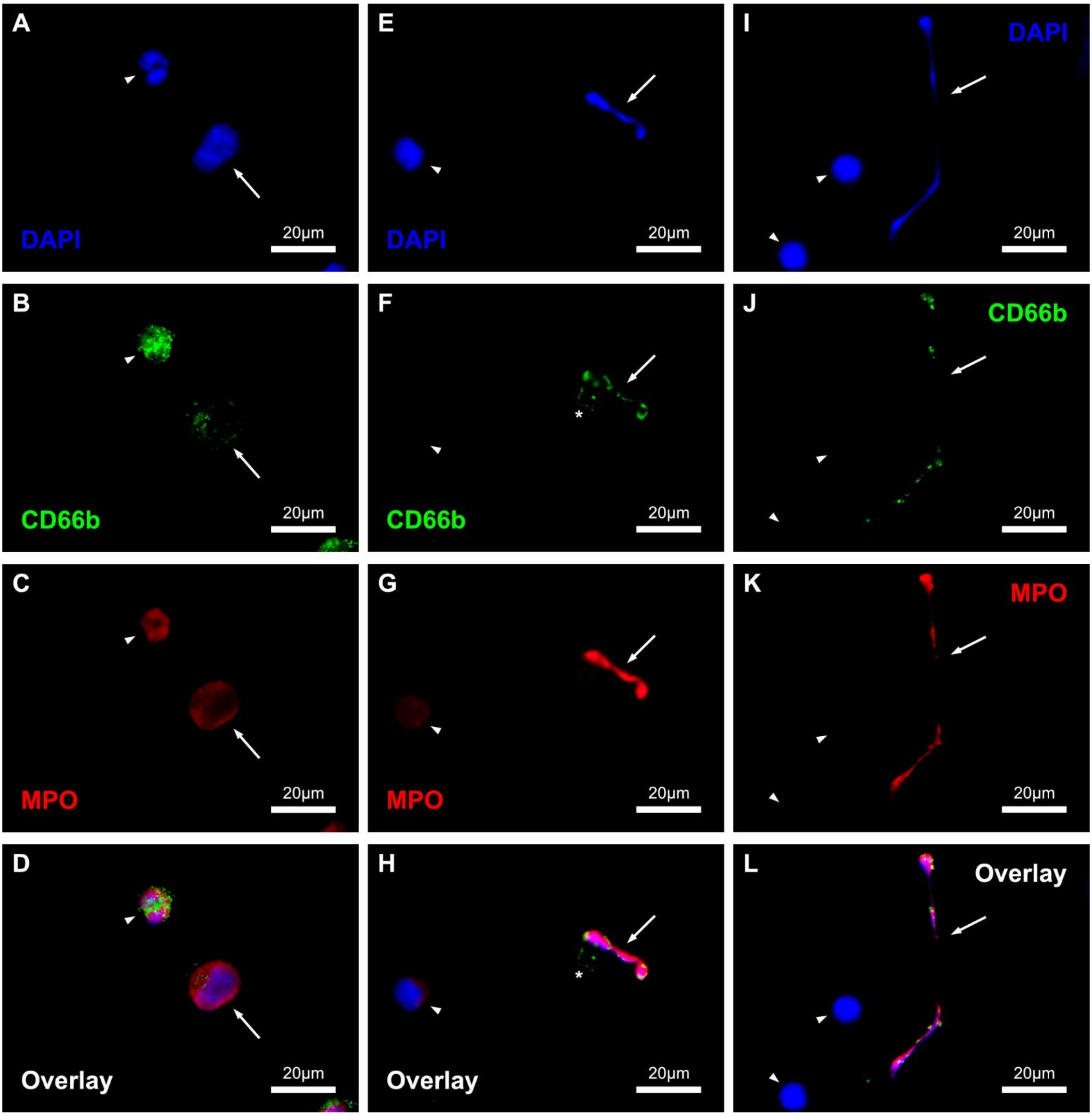

Supplementary Figure S6 Fluorescence microscopic images of the most common DNA-containing objects in blood smears from ECMO patients. Shown are the individual color channels of DAPI, CD66b and MPO staining, as well as the overlay of all channels. **A-D** show a neutrophil granulocyte (arrowhead) with typical lobulated nucleus and positive staining for both CD66b and MPO, together with a NET precursor (arrow) with large, decondensed nucleus, positive MPO, but markedly reduced CD66b signal compared to the granulocyte. Sections **E-H** present a monocyte (arrowhead) with detectable MPO, but without CD66b signal, and a NET filament (arrow) containing MPO and CD66b, as well as its residual cell body (asterisk), which can be identified by CD66b staining. **I-L** present two lymphocytes (arrowheads) with characteristic round nucleus without any detectable MPO or CD66b and a long, thin NET filament (arrow). Scale: 20µm (A-D).

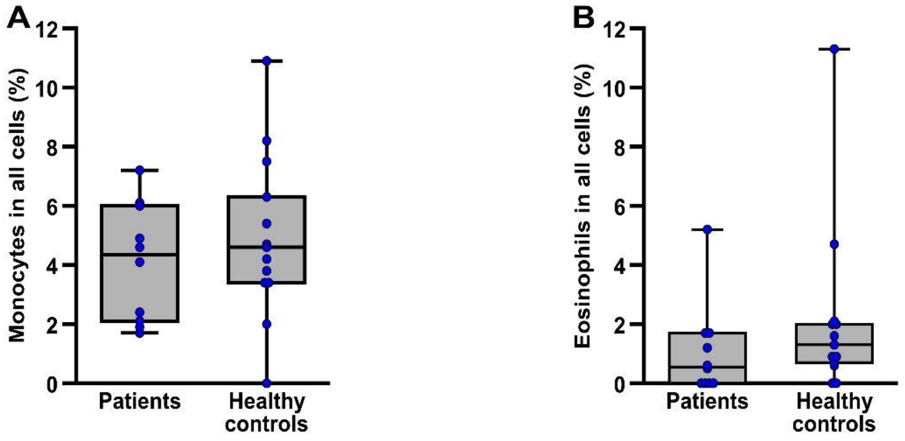

Supplementary Figure S7 Comparing the proportion of monocytes **(A)** and eosinophil granulocytes **(B)** among all detected cells in blood smears showed no significant differences between patients (n=10) prior to the beginning of ECMO therapy and healthy controls (n=13).

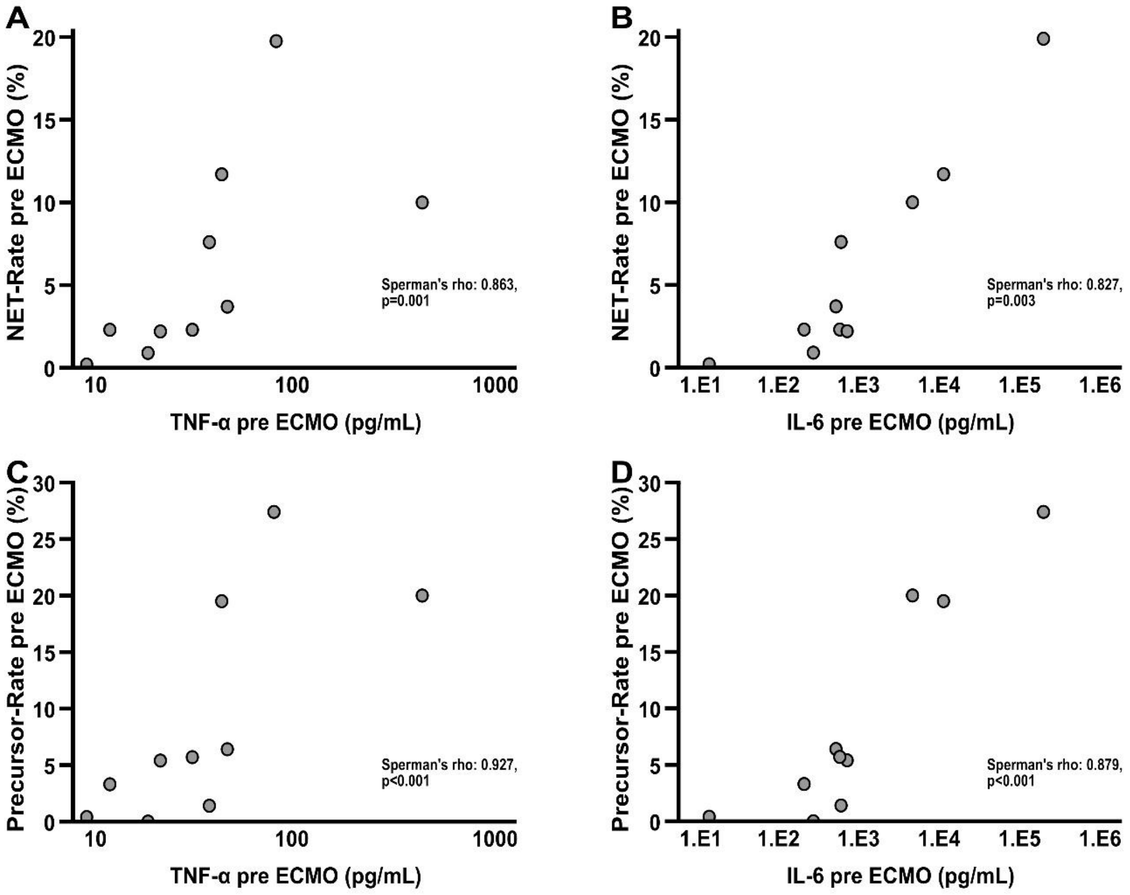

Supplementary Figure S8 A correlation of TNF-α **(A, C)** and IL-6 **(B, D)** levels with the NET **(A, B)** and precursor rate **(C, D)** before ECMO initiation revealed a significant association of cytokine levels and NET induction, which can also be seen in the corresponding scatterplots.

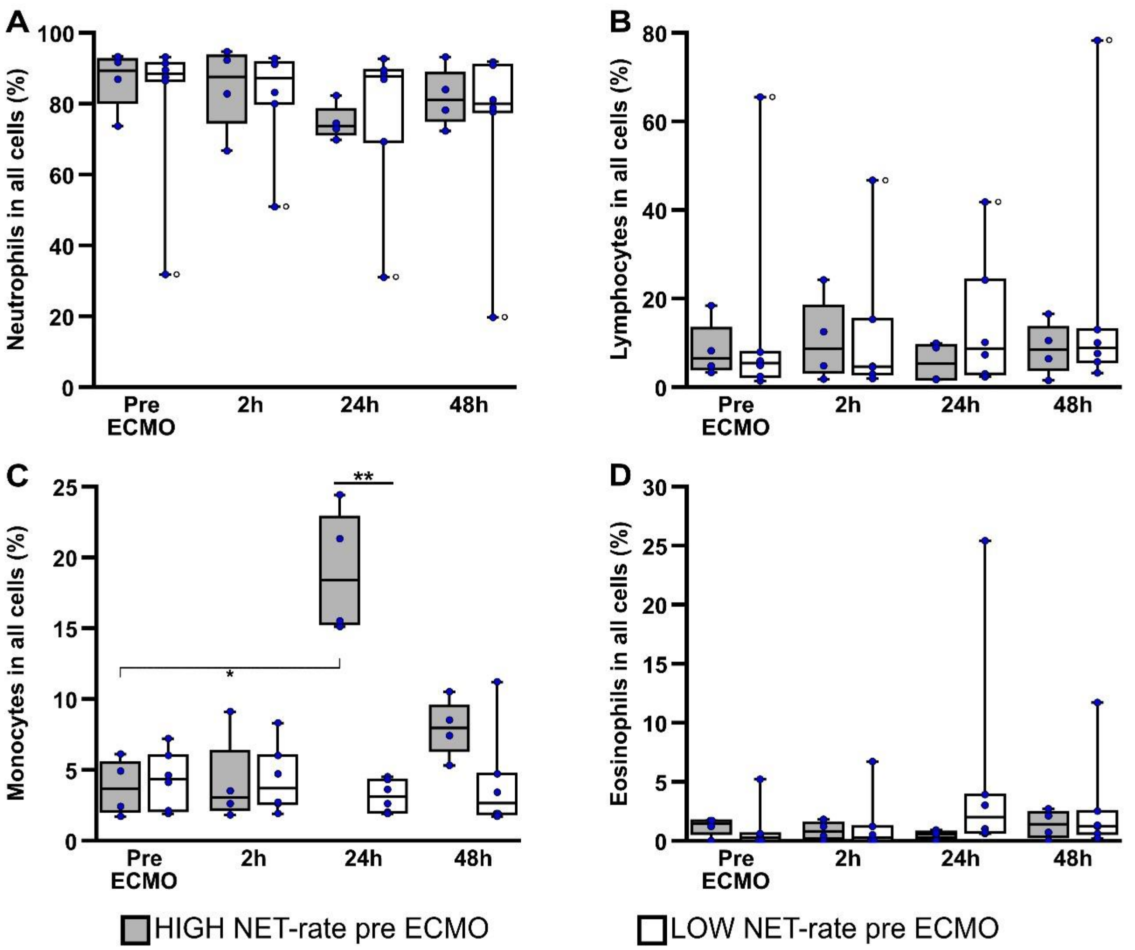

Supplementary Figure S9 Comparison of the proportions of the most frequent individual cell types among all detected nucleated cells in blood smears from patients (n=10) prior to the beginning and over the first 48h of ECMO therapy. Neutrophil granulocytes **(A)**, lymphocytes **(B)**, monocytes **(C)** and eosinophil granulocytes **(D)** were examined, taking into account patient groups defined by NET rate prior to the start of therapy. There were no significant differences except for a significant increase in detected monocytes in the HIGH-NET group 24 h after the start of therapy, which could not be observed in the LOW-NET group. The circle (o) marks the datapoints of one Patient suffering from chronic lymphocytic leukemia, resulting in a very high proportion of lymphocytes and a very low proportion of neutrophils in the blood smears. *p<0.05 and **p<0.01.

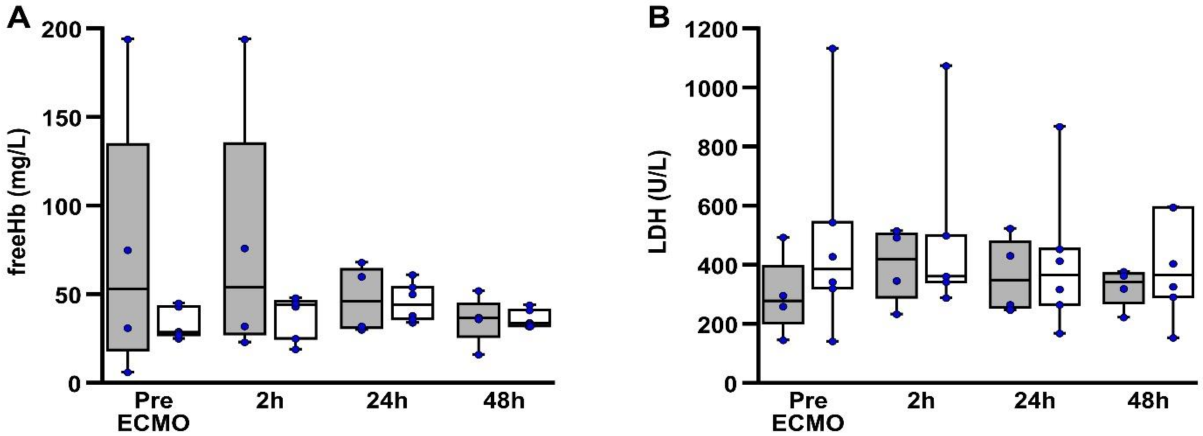

Supplementary Figure S10 Examination of free hemoglobin **(A)** and LDH **(B)** as indicators for hemolysis in the two patient groups defined by NET rate before initiation of therapy showed no significant differences over the first 48h of ECMO therapy.

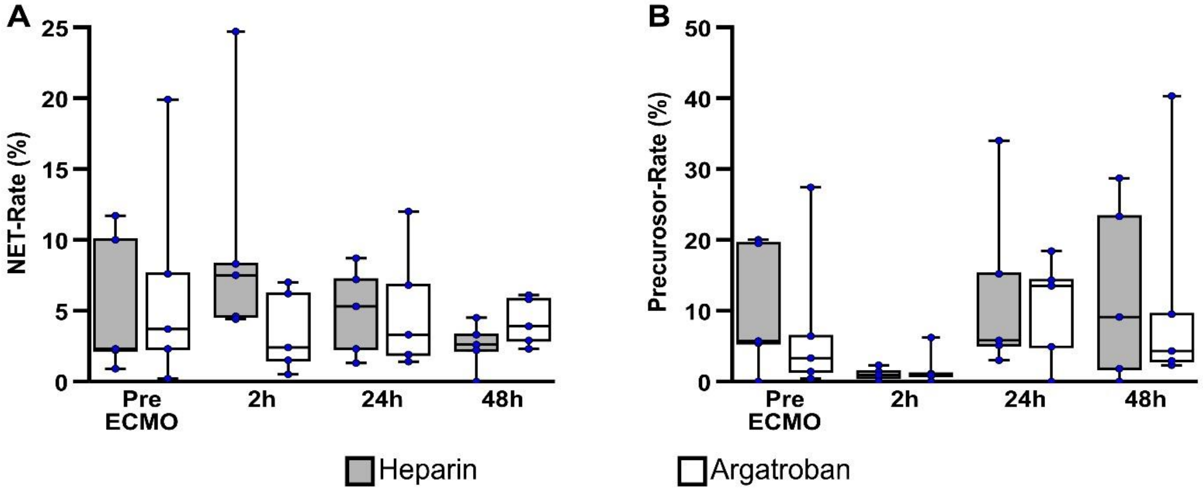

Supplementary Figure S11 Comparing NET **(A)** and precursor rates **(B)** of patients depending on anticoagulation, no significant difference was observed between heparin and argatroban.

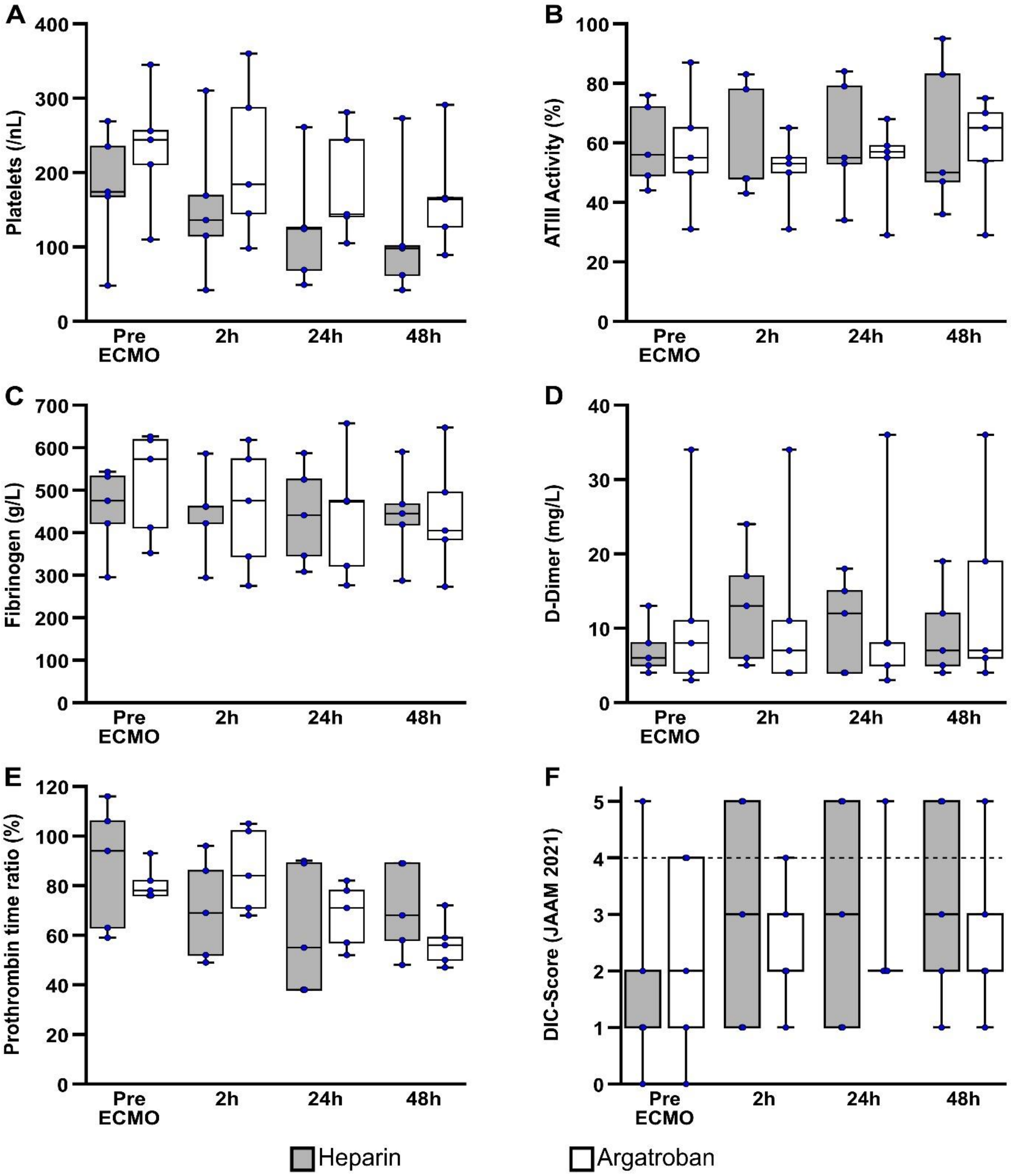

Supplementary Figure S12 A comparison of the coagulation parameters depending on the anticoagulation of the patients did not show any significant differences either. A slightly lower platelet count **(A)** was observed in patients treated with heparin, as described previously by Fisser et al.. However, the difference only appeared as a tendency, without reaching significance (p=0.222). AT III activity **(B)**, Fibrinogen **(C)** and D-Dimer **(D)** levels as well as prothrombin time ratio **(E)** and DIC-Score **(F)** showed no significant differences between the anticoagulation regimens.

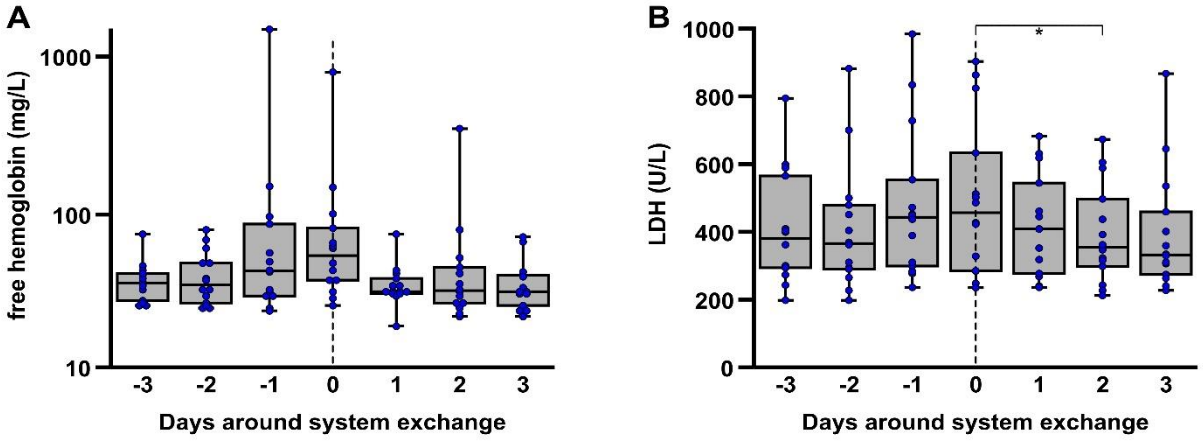

Supplementary Figure S13 Assessment of free hemoglobin **(A)** and LDH **(B)** as indicators of hemolysis in the collective of all ECMO system exchanges revealead a decreasing trend in free hemoglobin ($p=0.054$) as well as a significant LDH decrease ($p=0.011$) following the exchange. *$p<0.05$

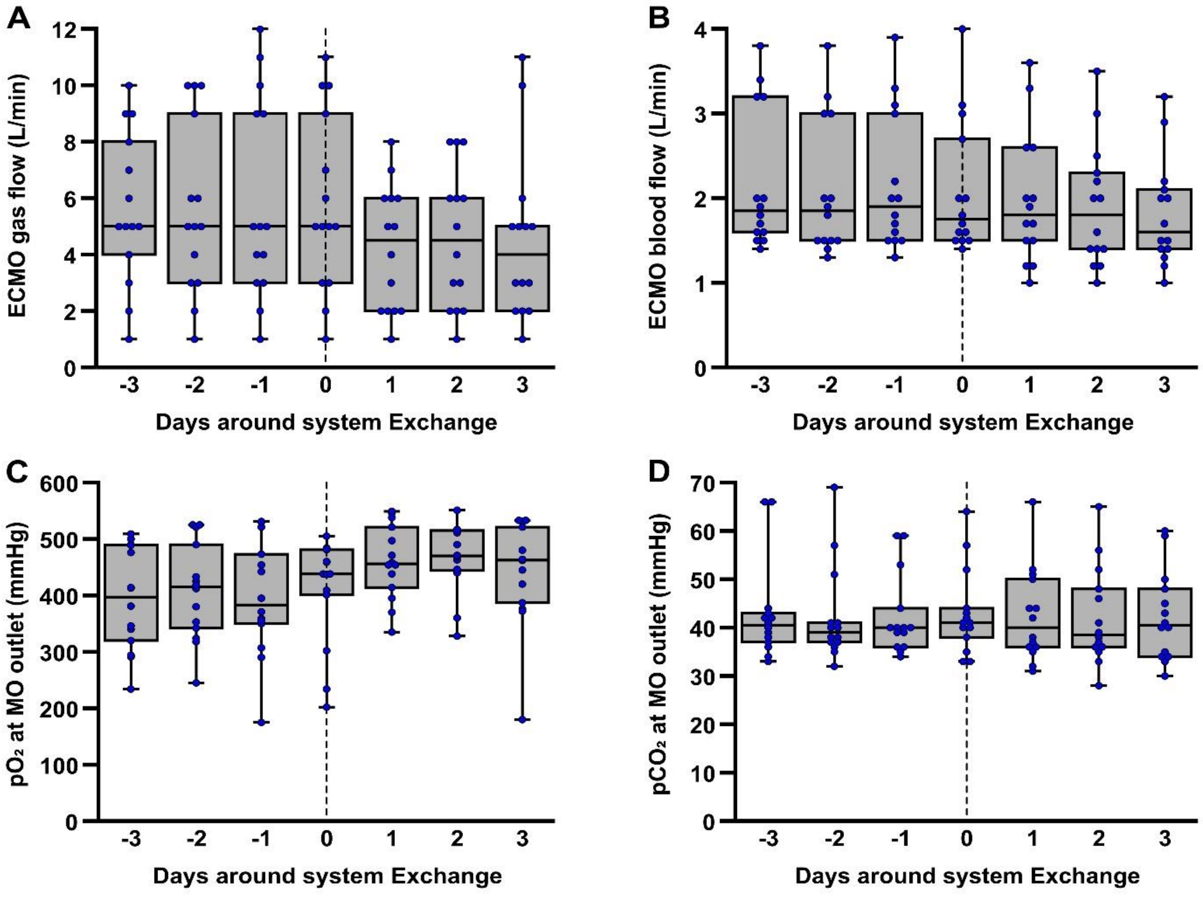

Supplementary Figure S14 Operational parameters of ECMO therapy around system exchanges. A significant ($p=0.031$) reduction in ECMO gas flow **(A)** was observed following the system exchange. ECMO blood flow **(B)** ($p=0.082$) und post-oxygenator pO_2 **(C)** ($p=0.268$) showed only trends but no significant differences. Post-oxygenator pCO2 **(D)** also showed no significant differences.

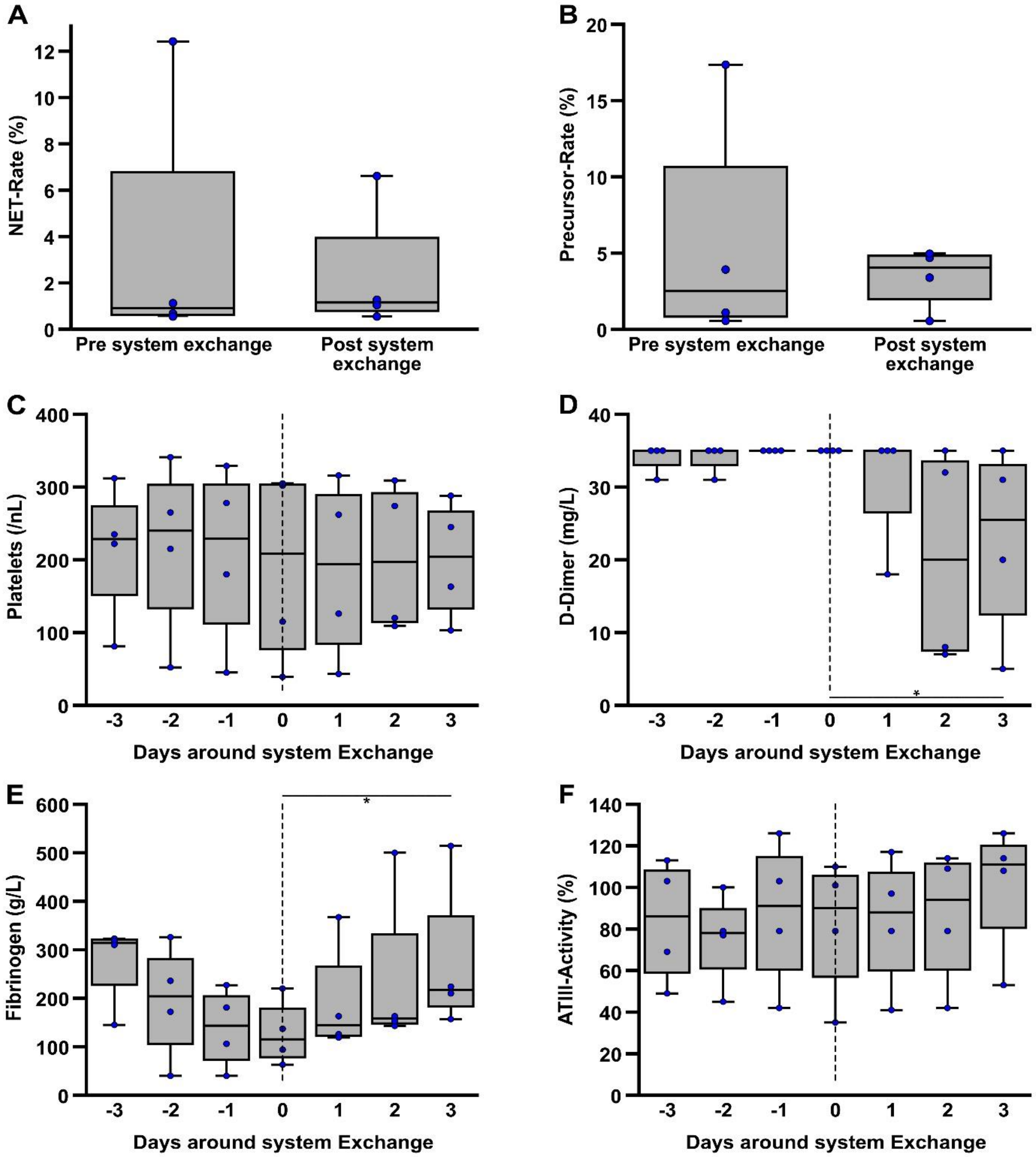

Supplementary Figure S15 Isolated analysis of the first ECMO system exchange in 4 patients requiring an replacement due to ECMO-associated complications (n=4) showed mostly consistent results compared to the pooled analysis of all System exchanges (Figure 5; NETs/ NET precursors: n=12; Coagulation parameters: n=14). In particular, NET **(A)** and precursor rates **(B)** before and after the first exchange showed no significant differences, however the limitations regarding the time of blood sampling mentioned in the main manuscript must be taken into consideration. The drop in fibrinogen levels **(E)** before a system exchange with a subsequent significant increase (p=0.038) as well as a significant drop in D-dimers **(D)** after a system replacement (p=0.028) could be observed to the same extent as in the pooled analysis. The slight increase in platelet counts after a system exchange witnessed in the pooled analysis could not be seen **(C)**, which, however, might be explained by the small sample size with relatively large dispersion. ATIII activity **(F)** presented no significant alterations over the period around a system exchange. *p<0.05
